# Nuclear pleomorphism in canine cutaneous mast cell tumors: Comparison of reproducibility and prognostic relevance between estimates, manual morphometry, and algorithmic morphometry

**DOI:** 10.1177/03009858241295399

**Published:** 2024-11-19

**Authors:** Andreas Haghofer, Eda Parlak, Alexander Bartel, Taryn A. Donovan, Charles-Antoine Assenmacher, Pompei Bolfa, Michael J. Dark, Andrea Fuchs-Baumgartinger, Andrea Klang, Kathrin Jäger, Robert Klopfleisch, Sophie Merz, Barbara Richter, F. Yvonne Schulman, Hannah Janout, Jonathan Ganz, Josef Scharinger, Marc Aubreville, Stephan M. Winkler, Matti Kiupel, Christof A. Bertram

**Affiliations:** 1 University of Applied Sciences Upper Austria, Hagenberg, Austria; 2 Johannes Kepler University Linz, Linz, Austria; 3 University of Veterinary Medicine Vienna, Vienna, Austria; 4 Freie Universität Berlin, Berlin, Germany; 5 The Schwarzman Animal Medical Center, New York, NY; 6 University of Pennsylvania, Philadelphia, PA; 7 Ross University School of Veterinary Medicine, Basseterre, St. Kitts; 8 University of Florida, Gainesville, FL; 9 Laboklin GmbH & Co. KG, Bad Kissingen, Germany; 10 IDEXX Vet Med Labor GmbH, Kornwestheim, Germany; 11 Heska, Loveland, CO; 12 Technische Hochschule Ingolstadt, Ingolstadt, Germany; 13 Hochschule Flensburg, Flensburg, Germany; 14 Michigan State University, Lansing, MI

**Keywords:** anisokaryosis, artificial intelligence, computer vision, dog, karyomegaly, mast cell tumor, mitotic count, nuclear pleomorphism, tumor heterogeneity

## Abstract

Variation in nuclear size and shape is an important criterion of malignancy for many tumor types; however, categorical estimates by pathologists have poor reproducibility. Measurements of nuclear characteristics can improve reproducibility, but current manual methods are time-consuming. The aim of this study was to explore the limitations of estimates and develop alternative morphometric solutions for canine cutaneous mast cell tumors (ccMCTs). We assessed the following nuclear evaluation methods for accuracy, reproducibility, and prognostic utility: (1) anisokaryosis estimates by 11 pathologists; (2) gold standard manual morphometry of at least 100 nuclei; (3) practicable manual morphometry with stratified sampling of 12 nuclei by 9 pathologists; and (4) automated morphometry using deep learning–based segmentation. The study included 96 ccMCTs with available outcome information. Inter-rater reproducibility of anisokaryosis estimates was low (k = 0.226), whereas it was good (intraclass correlation = 0.654) for practicable morphometry of the standard deviation (SD) of nuclear size. As compared with gold standard manual morphometry (area under the ROC curve [AUC] = 0.839, 95% confidence interval [CI] = 0.701–0.977), the prognostic value (tumor-specific survival) of SDs of nuclear area for practicable manual morphometry and automated morphometry were high with an AUC of 0.868 (95% CI = 0.737–0.991) and 0.943 (95% CI = 0.889–0.996), respectively. This study supports the use of manual morphometry with stratified sampling of 12 nuclei and algorithmic morphometry to overcome the poor reproducibility of estimates. Further studies are needed to validate our findings, determine inter-algorithmic reproducibility and algorithmic robustness, and explore tumor heterogeneity of nuclear features in entire tumor sections.

Variation in nuclear size and shape of neoplastic cells is an important histologic criterion of malignancy, and various evaluation methods have been used in previous studies. The most practical method is categorical estimation by pathologists, most commonly evaluating anisokaryosis or nuclear pleomorphism. Anisokaryosis is defined as the variation of nuclear size, and nuclear pleomorphism describes the variation in nuclear size and shape. Although these estimates have been shown to be relevant histologic prognostic factors for several tumors,^18,36,40,44,46^ some studies suggest low inter-rater and intrarater reproducibility.^22,47,48^ For both parameters, categories (mostly 3-tier such as mild, moderate, and severe) can only be vaguely defined, and applications of the same thresholds between pathologists may be problematic. Another limitation is that estimates are based on categories that need to be defined arbitrarily before conducting a study, and thresholds are not based on a statistical association with patient outcome.

Alternatives to estimates are computerized measurements of nuclear size and/or shape (nuclear morphometry) in digital images, which can be either done by pathologists using a measurement software (manual morphometry)^15,19,48^ or by image analysis algorithms (fully automated/algorithmic morphometry).^1,16,22^ Morphometry can be based on 2-dimensional measurements (nuclear area and shape)^22,33,51^ or 3-dimensional volume estimates based on stereological assumptions from 2-dimensional histologic sections using point-sampled intercepts (volume-weighted mean nuclear volume).^15,48^ Besides an assumed higher degree of reproducibility as compared with categorical estimates, morphometry enables the extraction of quantitative features from histological images and thereby creates more granularity and richness of the obtained data. A potential benefit of morphometry is the output of numerical values, which allows statistical determination of meaningful prognostic thresholds at the desired sensitivity and specificity trade-off. However, manual measurements by pathologists are time-consuming and, thus, difficult to conduct in a routine diagnostic setting. The number of nuclei measured was 100 in most previous studies on tumors.^5,15,33,37,48,49,51^ A time investment of 10 to 15 minutes has been reported for 75 and 166 (± 66 standard deviation [SD]) point-sampled intercept measurements,^15,48^ making this manual morphometry impracticable for routine diagnostic settings. In contrast, algorithms using state-of-the-art deep learning models are capable of eliminating human labor for these tasks and are very promising for quantitative evaluation of tumor markers.^11,22,39^ Fully automated nuclear morphometry can be done by post-processing of algorithmic nuclear segmentation (demarcation of all pixels representing the nuclei) masks (algorithmic output).^16^

Aside from morphometry based on nuclear segmentation, additional image analysis approaches have been used for automated evaluation of nuclear features: (1) image classification that categorizes images into tiers of anisokaryosis^35^ and (2) regression analysis that generates a continuous score based on the anisokaryosis tiers.^38^ Although all approaches achieve the goal of improving rater reproducibility by removing rater subjectivity, automated nuclear morphometry has several advantages as it is a quantitative and a very adaptable method. With 2-dimensional morphometry, several nuclear characteristics can be evaluated individually or in combination at any desired prognostic threshold, whereas classification and regression approaches are restricted to the predefined classes of morphological patterns. In addition, segmentation masks can be easily displayed as an overlay on the histological image, which allows visual verification of algorithmic performance to ensure reliability of the prognostic interpretation. Segmentation models can probably also be developed in a more consistent manner due to the nature of the ground truth data (accurate nuclear contour annotations) used for training. However, these data sets needed for the training of such segmentation models are more time-consuming to create when compared with the other approaches that require only 1 label (anisokaryosis class) per image.

Canine cutaneous mast cell tumors (ccMCTs) are one of the most pertinent skin tumors in dogs for possible application of these solutions due to their high frequency and malignant potential.^27^ Although studies on prognostic parameters for this tumor type are extensive in the veterinary oncologic literature, including the mitotic count (MC) and 2 multiparameter grading systems published in 1984 and 2011,^6,9,10,12,26,28,42,45^ further relatively inexpensive and practicable quantitative tests are needed to improve the prognostic ability of routine histopathologic assessment. Interestingly, despite their inclusion in the multiparameter grading systems (eg, karyomegaly and bizarre nuclei),^28,42^ the prognostic value of histologic estimates of nuclear characteristics has rarely been investigated in ccMCT.^14,50^ In an attempt to provide more objective criteria, the 2011 2-tier grading system defined a tumor as having karyomegaly if “the nuclear diameters of at least 10% of neoplastic mast cells vary by at least two-fold.”^28^ However, that definition of karyomegaly (origin of the word from ancient Greek for “large nuclei”) actually reflects anisokaryosis (variation in nuclear size), does not necessarily require abnormally enlarged nuclei, and would not be fulfilled when all neoplastic cells are karyomegalic. Furthermore, a significant association of karyomegaly as a solitary parameter with survival has not been shown to date.^14^ The prognostic value of manual nuclear morphometry of ccMCT has rarely been investigated for histologic^15^ and cytologic ccMCT specimens^51^ using either the 2- or 3-dimensional approaches. Fully automated solutions for nuclear morphometry have not been studied for ccMCT or any other tumors in domestic animals thus far, as opposed to tumors in humans.^1,16,54^ In addition, the variation of nuclear characteristics between different tumor areas in histological sections (tumor heterogeneity) has not been evaluated for ccMCT. This information on tumor heterogeneity is relevant for deciding optimal sampling strategies of regions of interest used for image analysis.

The primary objectives of this study are as follows:

- Explore the limitations of anisokaryosis estimates by pathologists, particularly regarding rater reproducibility.
- Develop nuclear morphometry methods that are feasible for a routine diagnostic setting, including practicable manual morphometry and automated morphometry.
- Investigate the measurement accuracy, reproducibility, and prognostic utility of the developed nuclear morphometry methods in comparison to the current gold standard morphometry method, anisokaryosis estimates, and the MC as an independent benchmark.

A secondary objective was to evaluate the heterogeneity of nuclear size in different tumor areas.

## Material and Methods

### Study Data Sets

Two separate sets of histological images of ccMCT with associated data were used in this study: (1) a data set with survival outcome and (2) a ground truth data set with ground truth annotations for outlines of tumor nuclei. The outcome data set was used to determine the reproducibility and prognostic value of the nuclear evaluation methods. The ground truth data set was primarily used to train, validate, and test the deep learning–based algorithm (fully automated morphometry) and was additionally used to evaluate the measurement accuracy of the nuclear evaluation methods. For both independent data sets, 1 representative tissue block for each ccMCT (all tumors had confirmed dermal involvement with possible subcutaneous infiltration) was selected, and histological slides were routinely produced using a section thickness of 2 to 3 µm and staining with hematoxylin and eosin. Digitization of the glass slides was done with the Pannoramic Scan II (3DHistech, Hungary) whole-slide image (WSI) scanner at default settings with a scan magnification of 400× (resolution of 0.25 µm/pixel). Each WSI represented a different ccMCT case. Using the software SlideRunner,^2^ a variable number of regions of interest (ROIs) within each WSI (1–2 for the ground truth data set, 3–5 for the outcome data set) were cropped and exported as TIFF files using lossless compression. Each ROI had a size of 0.1185 mm^2^ (equivalent to 0.5 standard high-power fields^39^) and an aspect ratio of 4:3. The ROI selection at low magnification was designed to include an arbitrary tumor region without paying particular attention to nuclear characteristics. This means that ROI selection was semi-random, except that tumor regions with widespread necrosis, severe inflammation and poor cell preservation, and nontumor regions were excluded, which was confirmed at high magnification once the region was selected. We decided against selecting a hotspot tumor location in this study to avoid any bias of oversampling regions with a particular nuclear characteristic (ie, over-representing a specific morphometric parameter). Selection of hotspot locations would have also hindered analysis of tumor heterogeneity.

The small ROI size was selected to ensure a quick (diagnostically practicable) computational time for algorithmic morphometry and to allow complete ground truth annotations (all neoplastic nuclei per image) of many cases. The variable number of selected ROIs for the 2 data sets (1–2 vs 3–5) is justified by the distinct requirements for the use of the data sets within this study. The total number and size of ROIs used for the ground truth data set were limited by the time investment required for the manual annotations. Considering this requirement for the ground truth data set, we tried to obtain a realistic variability in the ccMCT images for the ground truth data set by including a large number of different cases (and thus, a low number of relatively small ROIs per case). For the outcome data set, we wanted to obtain insight into the variability of nuclear parameters between different tumor regions (tumor heterogeneity) and its effect on prognostication for the outcome data set, and thus, a higher number of ROIs from different tumor locations were selected. The higher number of ROIs in the outcome data set was also used to account for low cellularity in a few cases and ensure that at least a few hundred nuclei were available for morphometry.

#### Outcome data set

The outcome data set consisted of 96 cases (1 tumor per patient) with known follow-up on patient survival. Histological sections were processed at Michigan State University (MSU) following routine protocols. Information on patient follow-up (date of surgery, date of death, and suspected cause of death based on the clinical interpretation of the patient) was collected through a survey sent to the submitters of the surgical tissue samples. Cases were excluded from the outcome population if the patients were lost to follow-up before 12 months after surgical excision of the ccMCT or were treated by systemic or radiation therapy, ie, all included patients were exclusively treated by surgical removal of the tumor. Following the routine trimming protocol at MSU and tumor margin evaluation (usually evaluating at least 4 peripheral margins and the deep margin), complete surgical excision was confirmed for all included cases. Postmortem examinations to verify the tumor burden were not available for any case.

For larger tumors (N = 91), 5 ROIs in different tumor locations at the periphery and center were selected. For smaller tumors, in which the 5 ROIs could not be placed without overlap (N = 5), the maximum number of nonoverlapping ROIs (3, N = 1; or 4, N = 4) was chosen.

#### Ground truth data set

ccMCT cases were retrieved from the diagnostic archives of 4 veterinary pathology laboratories (MSU, N = 21; The Schwarzman Animal Medical Center New York, N = 14; Freie University Berlin, N = 14; Vetmeduni Vienna, N = 15) with equal numbers of high-grade and low-grade tumors according to the 2011 histological grading system.^28^ For the samples from MSU, 2 ROIs per WSI were used, and for the samples from other laboratories, 1 ROI per WSI was used. As the cases from MSU were the target domain for application of the algorithm (see section “Outcome data set”), we decided to use 1 ROI from a central region and 1 ROI from a peripheral tumor region in an attempt to capture potential intratumoral variability. The other laboratories (with 1 ROI per case) were used to increase the robustness of the derived algorithm regarding laboratory-derived domain shift.

Using the software SlideRunner,^2^ 1 author (EP) delineated the contours of the nuclear membrane of all mast cells present in these 85 images using the polygon annotation tool. The final data set comprised 40 542 ground truth annotations (median per ROI = 455, range = 107–1049). The images were randomly assigned to the algorithm training subset (N = 61), the validation subset (N = 11), and the test subset (N = 13), whereas the 2 images per case from MSU were always assigned to the same training or validation subset. The test subset comprised 6111 annotations. This data set (images and ground truth annotations) was made publicly available for research purposes on https://git.fh-ooe.at/fe-extern/mastcell-data.

The annotations of the test subset were morphometrically measured (subsequently referred to as ground truth measurements) as listed in Table 1 and described for the gold standard manual morphometry below.

**Table 1.** List of parameters for manual and algorithmic morphometry evaluated in this study.

Feature	Measurement	Parameters
Size	Area (in µm^2^)	Mean, median, standard deviation (SD), 90th percentile (90th P), 90th P/median, mean of the largest 10% of the nuclei, percentage of large nuclei (>37.8 µm^2^ or >50.3 µm^2^), skewness (asymmetry of the data distribution)
Size	Eccentricity	Mean, SD, skewness
	Solidity	Mean, SD, percentage of nuclei with indentation (solidity <0.913), skewness

### Nuclear Evaluation Methods

For this study, the different methods of 2-dimensional nuclear size and shape evaluation investigated encompassed:

- Current routine method: anisokaryosis estimates by pathologists using 2 definitions:
  ○ 2-tier classification scheme (referred to as karyomegaly);
  ○ 3-tier classification scheme (referred to as 3-tier anisokaryosis).
- Current gold standard (benchmark) method: manual morphometry by a pathologist of at least 100 neoplastic nuclei (complete sampling of grids).
- Practicable alternative: manual morphometry of 12 representative neoplastic nuclei (stratified sampling).
- Automated solution: morphometry of all neoplastic nuclei segmented by a deep learning–based algorithm.

The methods of each test are specified in the following sections. Pathologists from 9 different laboratories conducted the prognostic tests and were blinded to the assessment by the other pathologists, to the results of the other prognostic tests, and to outcome information.

#### Anisokaryosis estimates

Eleven veterinary pathologists (TAD, C-AA, PB, MJD, AF-B, AK, KJ, RK, SM, BR, and FYS) participated in this study and were anonymized by a random identification number (P1–11). Anisokaryosis estimates were conducted at 2 time points with a wash-out time of at least 2 weeks. For time point 1 (11 participants), pathologists estimated the degree of anisokaryosis using 2 systems for the cases of the outcome data set. For time point 2 (9 participants), pathologists were instructed to estimate the degree of anisokaryosis a second time and to measure 12 neoplastic mast cell nuclei (manual morphometry) for each tumor in the test subset of the ground truth data set and the outcome data set. Images were provided to the pathologists through the online annotation platform EXACT.^34^ The images from the 3 to 5 ROIs of each case of the outcome data set were stitched to an image panel and separated by a black line to allow simultaneous viewing.

Two classification schemes (2- and 3-tier) for anisokaryosis estimation were applied by the pathologists for each case at 2 time points, resulting in 3840 data points for the outcome data set and 234 data points for the test subset of the ground truth data set. First, karyomegaly was assessed according to the definition given by Kiupel et al^28^ for the 2-tier MCT grading system, “nuclear diameters of at least 10% of neoplastic mast cells vary by at least two-fold,” as: (1) absent or (2) present. Second, the 3-tier anisokaryosis system consisted of the following categories: (1) none to mild, (2) moderate, and (3) severe variation in nuclear size of neoplastic mast cells. These stratifications were intentionally vaguely defined, as this is common practice in current veterinary literature.^17,18,29,31,40,41,43,47^ We also wanted to avoid creating arbitrary definitions of the 3 categories without known association with patient outcome.

#### Gold standard manual morphometry of ≥100 nuclei

Consistent with previous literature on nuclear morphometry in veterinary pathology,^5,33,37,49,51^ the gold standard morphometry method included manual annotations of at least 100 neoplastic mast cell nuclei. Although acknowledging that this test is too time-consuming for routine diagnostic application, we considered this method the benchmark for comparison with the other nuclear evaluation methods.

One pathologist (CAB) evaluated the images of the test subset of the ground truth data set and ROI 1 of the outcome data set by this method. In these images, a 5 × 6 grid overlay with thin black lines was added, ie, the images were separated in 30 equally sized grid fields. Using the software SlideRunner,^2^ the pathologist annotated as many grid fields (complete sampling) as needed to reach 100 nuclei. The sequence of grid fields selected followed a uniform meandering pattern that included the central grid fields first and the fields at the image borders last. Each grid field was completely annotated including each neoplastic nucleus that touched the grid borders and excluded nuclei that were cut-off at the image borders (Supplemental Figure S1). For the 109 evaluated images, an average of 108 annotations per image (minimum: 100, maximum: 133, total: 11,766) were created with an average of 7.7 of 30 grid fields (minimum: 2, maximum: 29). Time investment for the ≥100 annotations per image was 17.5 minutes on average (minimum: 14.5; maximum: 24).

From these manual annotations, different characteristics of the probability density function (Table 1) were calculated (morphometry). The nuclear area was defined by the number of pixels within the segmented nuclei and subsequent conversion into µm^2^ based on the scan resolution. The SD of the nuclear area reflects the variation of the nuclear size and thus was used as the primary parameter to compare with the 3-tier anisokaryosis estimates by pathologists. To approximate the 2-tier system definition for karyomegaly, “nuclear diameters of at least 10% of neoplastic mast cells [i.e., proportion of large nuclei] vary by at least two-fold [i.e., extent of nuclear size difference],”^28^ we evaluated 2 morphometric parameters. The proportion of abnormally enlarged (karyomegalic) cells was calculated by the number of nuclei above a case-independent reference size divided by the number of all tumor nuclei detected. The case-independent size for large nuclei was >50.3 µm^2^ (2 times the median area of all the annotated nuclei in the training/validation subset of the ground truth data set) or >37.8 µm^2^ (the 90th percentile of the annotated nuclei). We decided to select the thresholds for the karyomegaly definition from the ground truth data set (and not based on statistical correlation with the outcome data set) to avoid overfitting of the parameter on the outcome data set. The extent of nuclear size variation was determined by the 90th percentile divided by the median, representing the factor by which the largest 10% of nuclei, ie, potentially karyomegalic cells, differ from the median nuclear size of the same tumor (case-specific reference size). Instead of using the diameter according to the karyomegaly definition used in the 2-tier system, we based our calculations on the nuclear area, as an increase of the diameter is not proportional to the actual increase of nuclear size and a nuclear diameter is not representative of size for oval nuclei.

As indicators of nuclear shape, we measured eccentricity and solidity, as implemented in the scikit-image framework.^53^ The eccentricity measure is used to evaluate the roundness of the detected object. It is calculated by the ratio of the distance between the focal points of an ellipse over the major axis length. The closer this ratio is to 1, the more elongated the shape is. The closer the ratio is to 0, the more circular the shape is. For the calculation of the solidity measure, we used the ratio of the detected object area compared with the resulting convex hull of this area. A solidity value of 1 indicates that the detected pixel area has the same size as its convex hull. The closer this value is to 0, the more indentations are present and/or the larger the indentations are, corresponding to the bizarre nuclei definition of the 2011 grading system.^28^ Nuclear indentation thresholds evaluated were <0.913, <0.936, and <0.943, representing the 2nd, 5th, and 10th percentiles, respectively, of the training/validation subset of the ground truth data set. The percentage of indented nuclei over all detected nuclei was calculated. For calculation of the prognostic value of mean solidity, the direction of the values was inverted (ie, higher degrees of nuclear indentations are represented by larger values) by subtracting the mean solidity value of the 3 to 5 ROIs from 1 for each case.

#### Practicable manual morphometry of 12 nuclei

**Figure 1.**
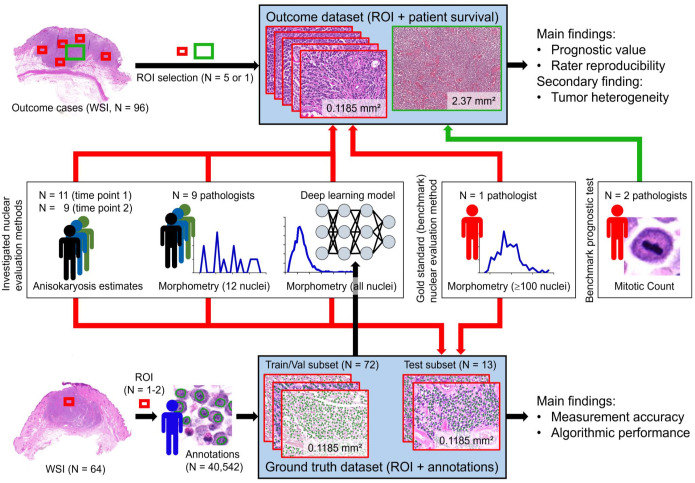
Overview of the study design. Two different sets of histologic images with associated data—the ground truth data set and the outcome data set were used to investigate different nuclear evaluation methods. ROI, region of interest; Train/Val subset, training and validation subset; WSI, whole-slide images.

At time point 2 (after anisokaryosis estimates), the 9 pathologists measured 12 neoplastic mast cell nuclei per case (Fig. 1). For cases with notable nuclear size differences, participants were instructed to perform stratified sampling with selection of 4 nuclei with a small, intermediate, and large area each. The relatively low number of nuclei was needed for practicability in routine diagnostics, whereas a stratified sampling method aimed at a representative frequency distribution. The images from the test subset of the ground truth data set were analyzed first (resulting in a total of 1407 annotations by 9 pathologists; in 3 instances, a participant had annotated 1 nucleus too many), and subsequently, the images of the outcome data set were evaluated (resulting in a total of 10 368 annotations). Pathologists were asked to use the 1 ROI of the outcome cases with the presumed (estimated) highest degree of anisokaryosis. The polygon annotation tool in Exact^34^ was used to outline the neoplastic nuclei. Based on the annotations, the mean, SD, and maximum nuclear area were calculated for each case and pathologist. We restricted the practicable manual morphometry to size parameters as pathologists reported difficulty in delineating the shape of the nuclei.

#### Automated morphometry using a supervised deep learning–based algorithm

We developed a Unet++-based segmentation model to create binary masks where pixels corresponding to the nuclei of the initial slide image are depicted as positive foreground (ones) in front of a negative background (zeros).^56^ We trained our model using the training and validation subsets of the ground truth data set. A more detailed description of the development method of the segmentation model is provided in the Supplemental Material. The developed model is available through https://git.fh-ooe.at/fe-extern/mastcell-data. As a post-processing step, we used connected component labeling provided by the scikit-image framework ^53^ to detect the individual nuclei within the segmentation mask. Based on the identified nuclei, we applied a filtering mechanism for objects smaller than approximately 7 µm^2^ to exclude segmented objects that do not represent complete and valid nuclei. Subsequent to segmentation and filtering of the individual mast cell nuclei, different morphometric parameters were calculated for each case (algorithmic morphometry) as listed in Table 1 and detailed in section “Gold standard manual morphometry of ≥100 nuclei”.

The developed algorithm was used to analyze the images of the test subset of the ground truth data set and the outcome data set. For the outcome cases, calculations were done for each ROI separately and averaged for all ROIs per case. Algorithmic segmentation resulted in 5794 objects for the test subset of the ground truth data set (average per image: 445, minimum: 75, maximum: 929) and 176 912 objects for the outcome data set (average per case: 1842, minimum: 604, maximum: 4090). Algorithmic morphometry was calculated from the uncorrected predictions, ie, no expert review of the segmentation masks was conducted in this study. However, for application of the algorithm in a diagnostic setting, we do recommend expert review of the segmented nuclei.

### Mitotic Count (Prognostic Value Benchmark) and Histologic Grade

As the prognostic value of the gold standard manual nuclear morphometry (using the 2-dimensional method) is not well established for ccMCT, the MC was determined as an independent benchmark with the intention of providing guidance for the interpretation of prognostic value of the nuclear evaluation methods. The MC is probably the single most important prognostic histological parameter for ccMCT with numerical values^10,27^ and is valuable in understanding how well a single prognostic test can theoretically discriminate patient survival for this specific outcome data set. This does not mean that the evaluated prognostic parameters must have a better prognostic value than the MC to be of relevance as cellular proliferation and nuclear pleomorphism reflect distinct malignancy characteristics of the tumor. We consider the histologic grading schemes^28,42^ to be less appropriate benchmark tests for this study because these systems combine nuclear characteristics (ie, karyomegaly and bizarre nuclei) with other morphologic criteria (such as the MC); thus, they are theoretically superior to a single morphometric parameter. As the grades are categorical values, some statistical tests for evaluation of the prognostic relevance are not possible as compared with numerical tests (such as the MC and nuclear morphometry), also hindering a thorough statistical comparison of the prognostic value with nuclear morphometry.

The MC was determined by 2 pathologists (first pathologist: CAB; second pathologist: TAD) in the images of the outcome data set according to current guidelines.^39^ First hotspot tumor regions were selected by the first pathologist, and subsequently, both pathologists annotated all mitotic figures in the same tumor regions. For region selection, the software SlideRunner^2^ with a plug-in for a rectangular bounding box overlay (4:3 ratio, area of exactly 2.37 mm^2^) was used, as previously described.^7^ This area box was placed in a mitotic hotspot location, which was selected based on the impression of mitotic activity evaluated by the pathologist in several tumor areas. Regions with widespread necrosis, severe inflammation, low tumor cell density, poor cell preservation, and extensive artifacts were excluded, if possible. Mitotic figures annotations (according to published definitions^20^) were done by both pathologists using SlideRunner ^2^ by screening these areas at high magnification twice to minimize the number of overlooked mitotic figures. The number of annotations per image represents the MC. For statistical analysis, we used the MC of each pathologist individually and the average of both pathologists (rounded up to a whole number).

The 2-tier grade was assigned by 1 pathologist (CAB) for the cases of the outcome data set according to the published criteria.^28^ The same MC as determined above by CAB was used for grading and the other parameters were determined in the WSIs, if the MC was below the threshold of the grading system. For both the MC and tumor grade, the pathologists were blinded to patient outcome and results of the nuclear evaluation methods.

### Statistical Analysis

Statistical analysis and graph creation were performed by GraphPad Prism version 5.0 (GraphPad Software, San Diego, California), IBM SPSS Statistics version 29.0 (IBM Corporation, Armonk, New York), and R version 4.2.2 (R Foundation, Vienna, Austria).

#### Rater reproducibility (outcome data set)

Rater reproducibility was determined for the cases of the outcome data set. For categorical estimates, inter-rater and intrarater reproducibility was determined by Light’s kappa and weighted Cohen’s kappa (k). The level of agreement was interpreted as poor = 0, slight = 0.01–0.20, fair = 0.21–0.40, moderate = 0.41–0.60, substantial = 0.61–0.80, and almost perfect = 0.81–1.00.^25^ For pathologists’ measurements (numerical values), inter-rater agreement was measured by the intraclass correlation coefficient (ICC; 2-way agreement, single measures, and random) with the following interpretation: poor = 0–0.39, fair = 0.40–0.59, good = 0.6–0.74, and excellent = 0.75–1.00.^25^ To compare the estimate categories assigned by each pathologist with the actual SD of nuclear area (algorithmic morphometry) of the individual case, linear regression was used.

#### Test accuracy (ground truth data set) and correlation (outcome data set)

The test accuracy of the nuclear evaluation methods was determined on 13 images from the test subset of the ground truth data set. Segmentation performance of the deep learning model (ie, overlap of the algorithmic segmentation map with the area of the ground truth annotations) was determined by the Dice score. The algorithmic performance to detect individual nuclei as compared with the ground truth was measured by the F_1_ score, recall (sensitivity), and precision. Measurement errors of algorithmic and manual measurements for the entire image were determined by comparison to the ground truth measurement using the root mean squared error (RMSE). Algorithmic and manual measurements and pathologists’ estimates were compared with the ground truth measurements using scatterplots. The correlation between the algorithmic morphometric parameters was analyzed on the outcome data set using Pearson’s method.

#### Prognostic value (outcome data set)

The outcome metrics primarily evaluated in this study (outcome data set) were tumor-related mortality at any time of the follow-up period and tumor-specific survival time. Dogs that died due to other causes (not considered ccMCT-related by the clinician) were grouped with cases that survived the follow-up period (receiver operating characteristic (ROC) curves, scatter plots, sensitivity, and specificity) or were censored (Kaplan-Meier curves and hazard ratios). The bias of this outcome metric is the variable follow-up period between cases without reported death (minimum of 12 months) and the lack of conclusive proof of the cause of death. It cannot be ruled out that tumor-related mortality was missed in a few cases with a relatively short follow-up period, while acknowledging that most ccMCT patients who survive the first 12 months will die from other causes. To eliminate the bias of a variable follow-up period and unproven cause of death, tumor-specific mortality and overall mortality within the first 12 months of the follow-up period were used as alternative outcome metrics. Overall death was defined as the occurrence of death regardless of the cause of death.

Numerical tests (algorithmic and manual morphometry and MC) were analyzed by ROC curves (plotting sensitivity against specificity for numerous thresholds) and the area under the ROC curve (AUC) with 95% confidence intervals (95% CIs). The distribution of the algorithmic measurements and the MC were displayed in scatter plots comparing cases with tumor-related mortality and other cases. Numerical tests with an AUC ≥0.700 were dichotomized by thresholds, resulting in a uniform sensitivity value for the different tests, which allows comparison of the associated specificity values. For the MC, the threshold proposed by Romansik et al^45^ was used to group cases with values of 0–5 and ≥6. The range of the morphometric measurements was divided into 200 intervals (thresholds increased by 0.5% steps), and 2 thresholds leading to a sensitivity of 76.9% (threshold 1: 10 true-positive cases and 3 false-negative cases; represents the sensitivity value for the MC determined by CAB) and 53.8% (threshold 2: 7 true-positive cases, 6 false-negative cases) were selected. If multiple cut-off values resulted in the desired sensitivity value, the highest value was picked.

With the categorical data (dichotomized numerical tests and pathologists’ estimates), Kaplan-Meier curves, hazard ratios with 95% CI (univariate Cox regression), as well as sensitivity (Sen, also known as recall), specificity (Sp), and precision (Pre, also known as positive predictive value), were calculated.

The pathologists’ measurements were analyzed individually, averaged, and combined (repeated measure data), when reasonable. For combined data, bootstrapping (AUC values) or a mixed model (Cox regression) was used to calculate 95% CIs.

#### Intratumoral heterogeneity (outcome data set)

Heterogeneity was evaluated for automated morphometric measurements between the different ROIs per case using the outcome data set. The difference between the 3 and 5 ROIs was determined by the coefficient of variation (SD/mean). The influence of the number of ROIs used for prognostic evaluation was determined by the AUC calculated from the mean measurements of 1, 2, 3, 4, and 5 ROIs based on their order of selection within the WSI. Tumor heterogeneity as a prognostic test was defined by the SD between the 3 and 5 ROIs and by the proportion of ROIs with a morphometric measurement above threshold 1 (defined as a hotspot) over all evaluated ROIs.

## Results

### Rater Reproducibility

Inter-rater reproducibility was slight to fair for estimates (time point 1) of karyomegaly (k = 0.226) and 3-tier anisokaryosis (k = 0.187), whereas it was good for practicable measurements (12 nuclei) of mean nuclear area (ICC = 0.637, 95% CI = 0.482–0.750), SD of nuclear area (ICC = 0.654; 95% CI = 0.577–0.730), and maximum nuclear area (ICC = 0.683, 95% CI = 0.603–0.756). Categorization of the practicable measurements (based on threshold 1 of the mean pathologists’ values) resulted in k = 0.432, k = 0.471, k = 0.497 for mean, SD, and maximum nuclear area, respectively. Consensus on the categories by at least 8/9 pathologists (excluding the 2 pathologists that did not do the measurements) occurred in 43 of 96 cases (45%) for karyomegaly estimates, in 62 of 96 cases (6465%) for mean nuclear area measurements, in 65 of 96 cases (68%) for SD of nuclear area measurements, and in 69 of 96 cases (72%) for maximum nuclear area measurements.

**Figure 2.**
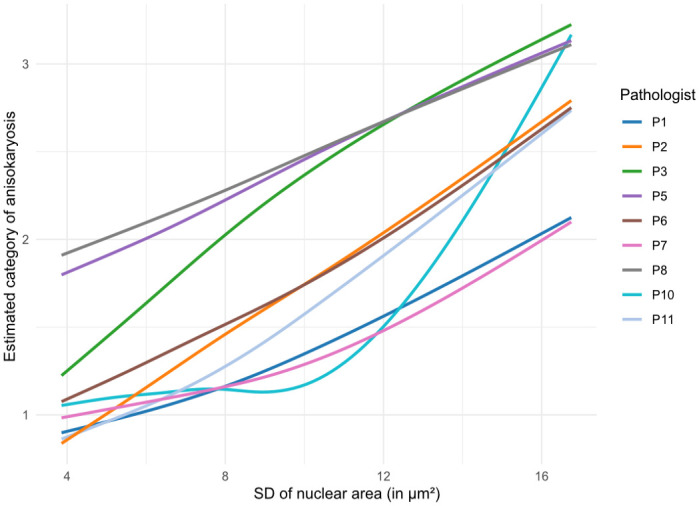
Illustration of the increase in the average 3-tier anisokaryosis estimate (time point 2, outcome data set) depending on the algorithmic standard deviation (SD) of the nuclear area for each pathologist (curves determined by linear regression). The curves show which anisokaryosis category was likely assigned by the corresponding pathologist to a case depending on the SD of nuclear area measured. Curves were smoothed using a spline regression. Pathologists P4 and P9 did not complete time point 2 and are not included in this graph. The results for anisokaryosis estimates at time point 1 are presented in Supplemental Figure S3.

Intrarater reproducibility between time points 1 and 2 was moderate for estimates of karyomegaly (k = 0.51; 95% CI = 0.44–0.58) and 3-tier anisokaryosis (k = 0.60, 95% CI = 0.55–0.65). Comparing inter-rater and intrarater reproducibility, the higher inter-rater inconsistency can be largely explained by different interpretations by the pathologists of the vaguely predefined thresholds. The variable application of the thresholds is illustrated in Fig. 2 and Supplemental Figure S3, which plot the 3-tier anisokaryosis categories to its corresponding algorithmically measured SD of the nuclear area.

### Test Accuracy and Correlation

Higher categories of karyomegaly and 3-tier anisokaryosis estimates were more commonly assigned to cases with higher SD of nuclear area based on the ground truth annotations (Supplemental Tables S1 and S2) or based on their own measurements of 12 nuclei (Supplemental Figure S5); however, there were marked inconsistencies between cases and pathologists.

The measurement errors/difference for gold standard manual morphometry to the ground truth measurements are provided in Supplemental Table S3. Comparison of the measurement errors/difference of the gold standard manual morphometry with the other morphometry methods revealed that the practicable manual method (12 nuclei) had a markedly higher degree of errors (Supplemental Tables S4 and S5 and Supplemental Figures S4 and S5) and algorithmic morphometry had a slightly higher degree of errors (Supplemental Table S6 and Figure S6). Overall, the measurement errors were lower for nuclear size parameters than for nuclear shape parameters.

**Figure 3.**
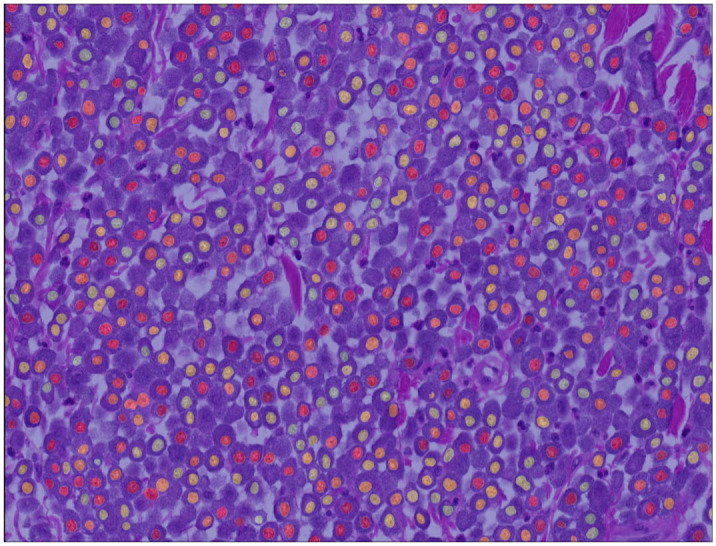
Example of an algorithmic segmentation mask for an outcome case. In rare instances, 2 nuclei are connected (undersegmentation). The algorithm has the tendency for omission of few neoplastic nuclei, whereas rarely false objects are detected.

Segmentation performance of the deep learning–based algorithm on the test subset of the ground truth data set had a Dice score of 0.785 (Fig. 3 and Supplemental Figure S7). The algorithm detected 5.794 individual neoplastic objects, which, compared with the annotations in the ground truth data set, resulted in a performance of F_1_ score = 0.854, recall = 0.830, and precision = 0.880. The model was able to adequately segment most tumor nuclei even in images with prominent metachromatic cytoplasmic granules, which partially obscured the nuclei (Supplemental Figure S8), and severe eosinophilic infiltration (Supplemental Figure S9). Comparison of the gold standard manual measurements with the algorithmic measurements on the outcome data set showed similar output of these methods for the nuclear size parameters but not for the evaluated nuclear shape parameters (Supplemental Figure S10).

Most morphometric parameters of nuclear area had a very strong correlation with each other including the 90th percentile to the median with a correlation coefficient of 0.936 (manual morphometry of ≥100 nuclei; Supplemental Table S7 and Figure S11) or 0.970 (algorithmic morphometry; Supplemental Table S8 and Figure S12). SD of eccentricity and solidity poorly to moderately correlated with the nuclear area parameters (SD, mean, median, 90th percentile, mean of the largest 10%, and percentage of large nuclei) with coefficients ranging between 0.100 and 0.320 for manual morphometry of ≥100 nuclei and between 0.404 and 0.674 for algorithmic morphometry.

### Prognostic Value

Of the 96 cases (78 low-grade cases and 18 high-grade cases) included in the outcome data set, death was attributed to the ccMCT in 13 cases with a median survival time of 4.3 months (range = 0.5–24). Other cases (N = 83) were lost to follow-up (N = 72) with a median follow-up period of 24 months (range = 12–45.3 months) or were reported to have died due to ccMCT-unrelated causes (N = 11) with a median survival time of 8.5 months (range = 0.2–29.7). At 12 months after surgical removal, 10 had died due to ccMCT-related and 6 due to ccMCT-unrelated causes. The demographic characteristics of this study population are described in the Supplemental Table S9.

**Figure 4.**
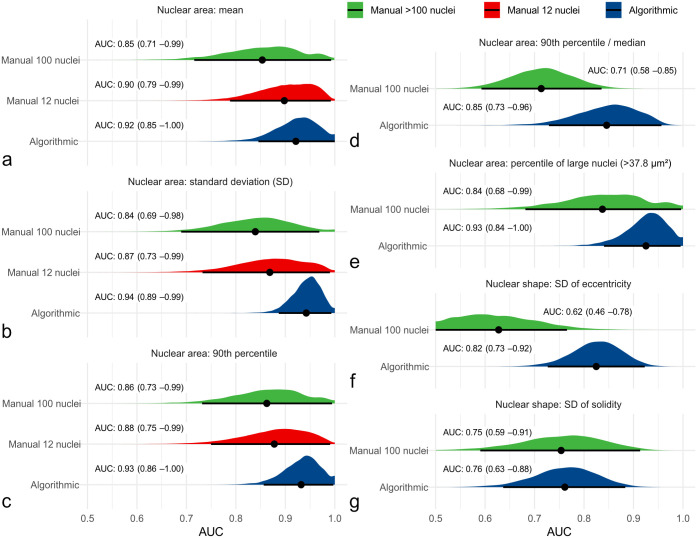
Graphical illustration of the area under the curve (AUC; point estimator indicated by black dot) with its 95% confidence intervals (black line) and probability density function (red, green, and blue areas) using bootstrapping for different nuclear morphometry parameters regarding tumor-specific survival. Algorithmic morphometry is displayed in blue and manual morphometry by pathologists in green (gold standard method) and red (practicable method) density functions. For practicable manual morphometry of 12 nuclei, the maximum value is equivalent to the 90th percentile. These graphs show that algorithmic and practicable manual morphometry have an at least equivalent prognostic value to the gold standard method regarding the AUC for all the displayed morphometric parameters, which include (a) mean nuclear area, (b) standard deviation (SD) of nuclear area, (c) 90th percentile of nuclear area, (d) 90th percentile/median nuclear area, (e) percentile of large nuclei with an area of >37.8 µm^2^, (f) SD of eccentricity, and (g) SD of solidity.

The frequency distribution of nuclear morphometry (Supplemental Figure S13) was able to discriminate cases without tumor-related and overall death from other cases as indicated by high AUC values for each method. In comparison to the gold standard nuclear morphometry (≥100 nuclei; Supplemental Tables S10–S12), practicable manual morphometry (12 nuclei; Supplemental Table S13) and algorithmic morphometry (Supplemental Tables S14–S16) achieved higher AUC values for patient survival (Fig. 4). For example, AUC (tumor-specific survival) of the SD of nuclear area was 0.839 (95% CI = 0.701–0.977) for gold standard morphometry, ranged between 0.823 and 0.960 for practicable morphometry of the individual pathologists and was 0.943 (95% CI = 0.889–0.996) for algorithmic morphometry. The independent benchmark MC had an AUC for tumor-specific survival of 0.885 (95% CI = 0.765–1.00, *P* < .001) for the first pathologists, 0.870 (95% CI = 0.767–0.972, *P* < .001) for the second pathologist, and 0.900 (95% CI = 0.807–0.993, *P* < .001) for the average count, which was somewhat below most morphometric size parameters of the practicable and algorithmic approach. On the contrary, algorithmic shape measurements had AUC values below the MC, whereas mean shape values did not provide any prognostic information (AUC values close to 0.5).

**Figure 5.**
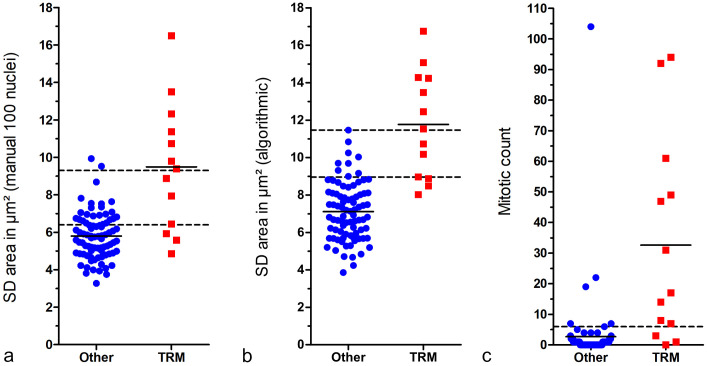
Scatterplots comparing cases with tumor-related mortality (TRM) with others (survived follow-up period or died due to tumor-unrelated cause). The short solid lines represent the mean of the measurement of the respective outcome group. The 3 graphs show that these prognostic factors can distinguish canine cutaneous mast cell tumor patients with TRM from others with high sensitivity or specificity, depending on the selected threshold. (a) Standard deviation (SD) of nuclear area measured by gold standard manual morphometry. The lower broken line represents threshold 1 (6.4 µm^2^; sensitivity: 76.9%, specificity: 71.1%) and the upper broken line represents threshold 2 (9.3 µm^2^; sensitivity: 53.8%, specificity: 97.6%). (b) SD of nuclear area measured by fully automated morphometry. The lower broken line represents threshold 1 (9.0 µm^2^; sensitivity: 76.9%, specificity: 89.2%) and the upper broken line represents threshold 2 (11.5 µm^2^; sensitivity: 53.8%, specificity: 100%). (c) Mitotic count by the first pathologist. The broken line represents the threshold according to Romansik et al^45^ (≥6; sensitivity: 76.9%, specificity: 92.8%).

Comparing the 2 outcome groups (tumor-related death vs. other) shows that the nuclear size measurements are able to distinguish patient outcome at a high sensitivity (threshold 1) or specificity (threshold 2), depending on the selected threshold (Fig. 5). Scatterplots for the further relevant parameters of manual (≥100 and 12 nuclei) and algorithmic nuclear morphometry are provided in Supplemental Figures S14–S16. The specificity and precision values based on threshold 1 are provided in Table 2 for manual and algorithmic SDs of area measurements, revealing a high performance of the algorithm and high variability between the pathologists in the threshold required to obtain the same sensitivity values. For SD of nuclear area based on algorithmic morphometry, a tumor-specific death rate of 3.9% (false omission rate) and 52.6% (precision) was determined for cases below and above the threshold of ≥9.0 µm^2^, respectively. Classification results of further morphometric methods and parameters are provided in Supplemental Tables S19–S22, and the variability of manual morphometry between pathologists is summarized in Supplemental Figure S17.

**Table 2.** Sensitivity, specificity, and precision regarding tumor-related mortality for the standard deviation (SD) of nuclear area measured by the different morphometric methods.

Method	Pathologist	Threshold	Sensitivity	Specificity	Precision
Manual (≥100 nuclei)	N/A	≥6.4 µm^2^	76.9%	71.3%	29.4%
Manual (12 nuclei)	P1	≥8.0 µm^2^	76.9%	84.3%	43.5%
	P2	≥9.8 µm^2^	76.9%	84.3%	43.5%
	P3	≥10.9 µm^2^	76.9%	88.0%	50.0%
	P5	≥13.4 µm^2^	76.9%	86.7%	47.6%
	P6	≥8.5 µm^2^	76.9%	69.9%	28.6%
	P7	≥12.2 µm^2^	76.9%	95.2%	71.4%
	P8	≥10.5 µm^2^	76.9%	84.3%	43.5%
	P10	≥14.0 µm^2^	76.9%	90.4%	55.6%
	P11	≥12.2 µm^2^	76.9%	84.3%	43.5%
Algorithmic	N/A	≥9.0 µm^2^	76.9%	89.2%	52.6%

N/A, not applicable; P1–P11, pathologists 1–11.

The classification threshold is adapted equally for all tests to the sensitivity value of the mitotic count by one of the 2 pathologists (CAB) at the proposed threshold by Romansik et al^45^ of ≥6 (sensitivity: 76.9%; specificity: 92.8%; precision: 62.5%).

**Figure 6.**
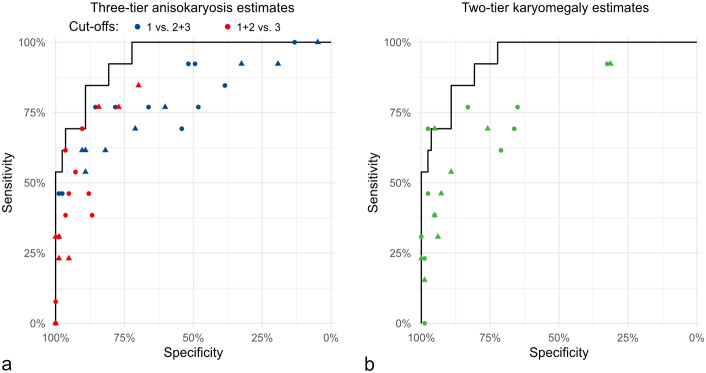
Comparison of the pathologists’ sensitivity and specificity values for anisokaryosis estimates of time points 1 (dots) and 2 (triangle) regarding tumor-related mortality. The solid line in both graphs represents the same ROC curve for standard deviation (SD) area measured by the deep learning-based algorithm. (a) Pathologists’ estimates (dots and triangles) based on the 3-tier anisokaryosis (1: none to mild, 2: moderate, or 3: severe) approach. Two of the 3 categories are combined into none to moderate vs severe (red symbols) and none to mild vs moderate to severe (blue symbols) anisokaryosis. (b) Pathologists’ estimates by the karyomegaly definition (green symbols). Both graphs show that anisokaryosis estimates by all pathologists have a good prognostic value almost reaching the performance of the deep learning–based algorithm (ROC curve). However, the individual pathologists’ sensitivity and specificity values vary markedly, indicating relevant differences in the thresholds between the categories that each pathologist applies.

The categorical anisokaryosis estimates by pathologists resulted in highly variable Sen and Sp values for tumor-specific survival ranging between Sen = 100%/Sp = 4.8% (pathologist 8) to Sen = 46.2%/Sp = 98.8% (pathologist 7) for anisokaryosis 1 vs 2 and 3, between Sen = 84.6%/Sp = 69.9% (pathologist 5) to Sen = 0%/Sp = 100% (pathologist 1) for anisokaryosis 1 and 2 vs 3, and between Sen = 92.3%/Sp = 31.3% (pathologist 11) to Sen = 0%/Sp = 98.8% (pathologist 5) for karyomegaly (Fig. 6 and Supplemental Tables S17 and S18). The performance of the estimates by almost all pathologists was below the algorithmic ROC curve (Fig. 6). As compared with the MC of the first pathologist classified by the threshold proposed by Romansik et al^45^ (Sen = 79.6%, Sp = 92.8%), the sensitivity for tumor-specific survival was slightly higher for the 2011 2-tier histologic grade (Sen = 84.6%, Sp = 91.6%) due to the combination with the nuclear characteristics. The MC classified by the threshold proposed by Romansik et al^45^ of the second pathologists resulted in a Sen of 84.6% and Sp of 75.9% and of the average count resulted in a Sen of 76.9% and Sp of 85.5%.

**Figure 7.**
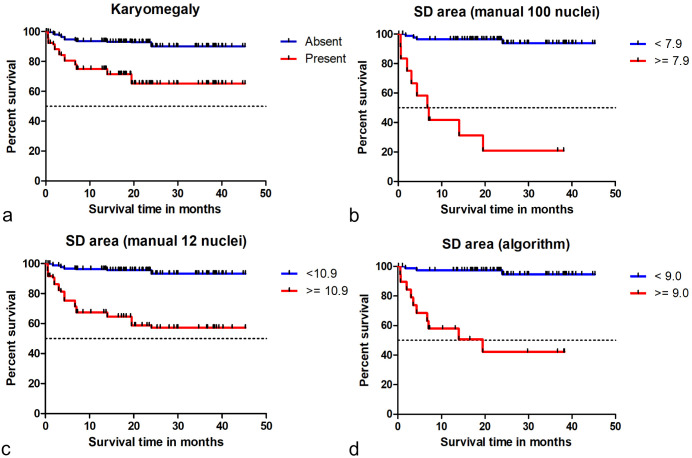
Kaplan-Meier curves regarding tumor-specific survival time for different tests on nuclear size evaluation. (a) Karyomegaly estimates (combined data from time point 1 of all 11 pathologists). The hazard ratio is 7.6 (95% CI = 5.7–10.1, *P* < .001). (b) Standard deviation (SD) of nuclear area measured by gold standard manual morphometry (≥100 nuclei). The hazard ratio for this test is 24.8 (95% CI = 7.5–81.2, *P* < .001). (c) SD of nuclear area measured by practicable manual morphometry (12 nuclei; combined data of all 9 pathologists). The hazard ratio for this test is 9.0 (95% CI = 6.0–13.4, *P* < .001). (d) SD of nuclear area measured by algorithmic morphometry. The hazard ratio for this test is 18.3 (95% CI = 5.0–67.1, *P* < .001). These graphs show that the 4 prognostic factors can separate patients (based on the proposed threshold) according to their survival probability.

Kaplan-Meier curves and hazard ratios determined that patient survival time was significantly different for cases with low vs high anisokaryosis based on the predefined categories of the estimates and based on threshold 1 for nuclear morphometry of SD of area (Fig. 7, Supplemental Figures S18–S22, and Supplemental Tables S23–S26). The hazard ratios of the morphometric size measurements were higher than those for the shape measurements (Supplemental Tables S24 and S26). For the MC (classified by the threshold proposed by Romansik et al^45^), the hazard ratios were 30.5 (95% CI = 7.8–118.0, *P* < .001) for the first pathologist, 13.8 (95% CI = 3.0–62.6, *P* < .001) for the second pathologists, and 14.6 (95% CI = 3.9–53.4, *P* < .001) for the average count (Supplemental Figure S23). The histologic grading system resulted in a hazard ratio of 46.5 (95% CI = 9.6–223.3, *P* < .001).

### Intratumoral Heterogeneity (Algorithmic Morphometry)

Some variability in the measurements between the tumor locations of the 3 to 5 ROIs was noted for the different algorithmic morphometric parameters (Supplemental Table S27 and Figure S24). Regarding the prognostic classification based on threshold 1 (the sensitive threshold) of SD of the nuclear area, 39 of 96 cases (41%) had divergent (low and high) measurements between the individual ROIs (Table 3). Based on threshold 2 (the specific threshold), divergent classification between the ROIs of 1 tumor occurred in 18 of 96 cases (19%). However, the overall test performance (determined by the AUC) only increased mildly with the number of ROI used for statistical analysis, except for SD of eccentricity (Supplemental Figure S25).

**Table 3.** Distribution of the outcome of cases based on the proportion of hotspot regions of interest (ROI, classified by threshold 1) according to the standard deviation of nuclear area measurements (fully automated morphometry; calculated by the number of ROI with a measurement above threshold 1 (9.0 µm^2^) divided by the number of evaluated ROI), comparing cases with tumor-related mortality (TRM) to other cases without TRM.

Outcome	Proportion of hotspot ROI						
	0/3–5 (0%)	1/5 (20%)	2/5 (40%)	2/4 (50%)	3/5 (60%)	4/5 (80%)	5/5 (100%)
TRM	1	0	3	0	0	2	7
Other	47	18	6	1	6	3	2
Death probability	2%	0%	33%	0%	0%	40%	78%

For each case, 3 (N = 1), 4 (N = 4), or 5 (N = 91) ROIs were analyzed.

Tumor heterogeneity as a prognostic test had a high AUC value for tumor-related death (Supplemental Table S28), ie, cases with a higher number of ROIs above the prognostic threshold or cases with high variability between the different tumor locations were more likely to be aggressive tumors. For the morphometric parameter of SD of nuclear area, the SD between the different ROIs had an AUC of 0.790 (95% CI = 0.632–0.947), and the proportion of hotspot ROIs (above threshold 1 = 9.0 µm^2^) had an AUC of 0.890 (95% CI = 0.780–1.00). With a higher proportion of hotspot ROIs, the tumor-related death probability increases (Table 3), and grouping cases with 0%–20% vs ≥40% hotspot ROI resulted in a Sen of 92.3% and Sp of 78.3%.

## Discussion

This study assessed several nuclear evaluation methods, including anisokaryosis estimates, manual morphometry, and algorithmic morphometry pertaining to reproducibility, measurement accuracy, and prognostic utility. This work represents a comprehensive veterinary study to: (1) compare anisokaryosis estimates with morphometry and (2) develop and validate (2A) a practicable manual morphometry method and (2B) algorithmic morphometry. The results of this study highlight the limitations of estimating nuclear characteristics of tumor cells as part of prognostic histologic evaluation, particularly regarding rater reproducibility. Nuclear morphometry has several advantages over categorical evaluation of nuclear features; however, large numbers of cells cannot be measured by pathologists in a routine diagnostic setting. Measurement of ≥100 nuclei (gold standard method) required an average of 17.5 minutes. Our proposed practicable manual nuclear morphometry and automated nuclear morphometry approaches can overcome these limitations while having a high prognostic value and practicability that allows integration into routine diagnostic workflows of laboratories using digital microscopy. The choice of using small ROIs for algorithmic morphometry was mostly made to ensure practicability of the proposed approach, including the need for computational resources for image analysis. The size of the ROIs is roughly equivalent to photomicrographs at 400× using a camera mounted on a light microscope, making this approach also feasible for pathologists that do not have access to WSI.

The results of this study show low inter-rater reproducibility for anisokaryosis estimates in ccMCT. Given the wide variation in sensitivity and specificity (tumor-specific survival) when comparing individual pathologist’s estimates, this parameter has different prognostic values between pathologists. Low inter-rater reproducibility may be the result of the following 2 issues: (1) distinct anisokaryosis categories are difficult to precisely define and (2) the defined thresholds between the categories are interpreted differently by individual pathologists. A vague definition of the categories (ie, mild, moderate, severe, or similar) is common in the current literature;^17,18,29,31,40,41,43,47^ thus, we decided to use a similar approach for the 3-tier anisokaryosis method. Other studies provide a more specific definition for the anisokaryosis categories with percentage of affected cells and/or degree (fold-change) of nuclear size variation,^21,28,36^ as for the karyomegaly definition used in this study. Regardless of the use of a vague or more specific definition, study participants had difficulty applying the categories in the same manner. The specific definitions (fold-change size variation in X% of nuclei) are interpreted differently by pathologists when estimated. Further studies are needed to evaluate methods that improve reproducibility, such as pictorial illustrations of each category or reference sizes within the image based on a scale overlay (digital microscopy) or nontumor cell within the image (light microscopy), similar to that used for the lymphoma subtype classification.^52^ Of note, average neoplastic mast cells in the tumor section seem to be a less ideal reference size, as aggressive tumors not only have a higher variation in nuclear size but also a higher mean/median nuclear area. When creating new definitions for reproducible anisokaryosis estimates, the numerical morphometry data may guide finding prognostically meaningful thresholds a priori.

Low reproducibility of pathologists’ estimates is the main motivation for morphometry^15,48^ and the development of the different algorithmic approaches. Manual morphometry has been shown to have high reproducibility,^15^ but it is impractical to measure a larger number of cells for routine diagnostic pathology. In our study, we limited the number of cells to 12 (stratified sampling method) with the intention of creating a practical quantitative test, although some study participants commented that they found annotating the 12 nuclei challenging for the time available in a diagnostic setting. Despite improved reproducibility as compared with estimates, significant differences between pathologists were still noted, suggesting that 12 selected nuclei with stratified sampling may not be representative enough for each case. Interestingly, the maximum nuclear area measured by manual morphometry (12 nuclei) had the highest inter-rater reproducibility in this study (compared with anisokaryosis estimates and other parameters of practicable manual morphometry), while also having a good prognostic value. Measuring just one of the largest neoplastic nuclei in the tumor section would improve the feasibility of applying morphometry and should be evaluated as a prognostic factor in future studies. Owing to intratumoral heterogeneity, reproducibility may be reduced when this task is performed on WSI and not restricted to a few preselected tumor regions. Future studies should compare reproducibility of manual morphometry depending on the number of tumor nuclei annotated (less or more than 100 nuclei) and the method of cell selection (complete vs stratified). Using 1 algorithm will result in 100% reproducibility;^11^ however, the reproducibility between different segmentation models (based on different network architectures and/or training/validation data) that may be applied in different laboratories needs to be evaluated (interalgorithmic reproducibility).

Identification of nuclei and their outlines is generally a straightforward task for pathologists and trained deep learning models. However, it is our experience from the manual annotations (pathologists’ experiments and creation of the ground truth data set) that identification of the cell type (neoplastic mast cell vs stromal cell, etc) and nuclear borders can be difficult for some cells. Nuclear membranes may be obscured in heavily granulated ccMCT. We therefore evaluated the measurement accuracy of manual and algorithmic morphometry. Compared with the ground truth, manual morphometry of 12 nuclei resulted in an overestimation of nuclear size, suggesting that pathologists had a tendency to oversample larger cells within the ROIs. In contrast, the algorithm sampled most nuclei in the ROIs (little sampling bias) and was able to accurately segment mast cell nuclei, resulting in a smaller error for nuclear size measurements. The perceived sources of errors of the segmentation model are discussed in the following sentences. Undersegmentation (algorithmic division of the image into too few segments, ie, nuclei, leading to the interpretation of excessively large nuclei) was a rare problem that resulted in slight overestimation of karyomegalic cells. As compared with other tumor types, undersegmentation may be less relevant due to the lack of close cell contact between the neoplastic round cells and the moderate amount of cytoplasm that separates the neighboring mast cell nuclei.^22,24^ Oversegmentation (segmentation of only a part of the nucleus) and omission of neoplastic cells rarely occurred, particularly when cytoplasmic granules obscured the nucleus. It should be noted that the omission of neoplastic cells does not affect the overall morphometry if this is a random error. Another source of error that rarely occurred for our algorithm is the segmentation of non-neoplastic nuclei, such as stromal cells or endothelial cells, which should be included in higher numbers in future training data sets. It was beyond the scope of the present study to evaluate the robustness of the segmentation models to changes in the image characteristics, particularly the different WSI scanners and tumor types, as these have been identified as relevant sources of a domain shift that lead to reduction in the algorithmic performance of deep learning–based mitotic figure algorithms.^3,4^

All these sources of algorithmic error—ie, undersegmentation, false object localization, and potential domain shift—may bias the nuclear size measurements, and further improvements of the model are warranted. The potential sources of domain shift (particularly different WSI scanners) should be considered for the development of future data sets. Deep learning–based models are the state-of-the-art for nuclear segmentation (as compared with traditional machine learning methods) as demonstrated by several computer science challenges.^23,30^ Future studies should evaluate an instance segmentation model for ccMCT that can better separate overlapping/connected nuclei and completely segment or omit obscured nuclei. Based on the frequency and extent of error in nuclear segmentation, different degrees of human–machine interaction (computer-assisted vs fully automated prognosis) may be recommended to reduce errors during case evaluation.^7,39^ In this study, we did not apply any human–machine interaction; however, we suggest that the model’s segmentation mask (overlay on the hematoxylin and eosin image) should be verified visually by a trained pathologist if algorithmic morphometry is applied for routine diagnostic service. This transparency of the intermediate results of the segmentation model can be used to remove algorithmic errors, including undersegmentation and oversegmentation as well as detection of non-neoplastic nuclei.

Two-dimensional morphometric measurements were employed in the present study. Other studies have used stereological estimates of nuclear volume (3-dimensional).^15,48^ The rationale for estimating nuclear volume is that the measured area of the nucleus (a 3-dimensional structure) in 2-dimensional tissue sections is influenced by the position and orientation of the nucleus to the plane of section.^13,15^ The incorrect assumption of orderly positioning and orientation of the nuclei along the plane of section introduces bias, such as the increased chance of evaluating larger nuclei more frequently.^13^ While we acknowledge that our measurements may not perfectly correlate with 3-dimensional nuclear characteristics, we assume that our measurements follow statistical principles that allow calculation of the probability density function. We argue that the 3-dimensional methods might not be ideal either. Calculations of a 3-dimensional volume from a 2-dimensional area measurement is based on the assumption that all 3-dimensional structures have the same volume, whereas the difference in the measured area is assumed to be related to the different planes of sections through the nuclei. However, a uniform nuclear volume is not the case for neoplastic nuclei with anisokaryosis. The benefit of 2-dimensional morphometry is that different parameters (mean, SD of nuclear area, 90th percentile, etc) of size and shape can be evaluated, whereas the stereologic approach is restricted to the mean volume. A direct comparison of the prognostic value of 2- and 3-dimensional-based methods may be an interesting subject for future studies.

Most nuclear size parameters had good prognostic value, while practicable manual and algorithmic morphometry predicted outcomes a little bit better than gold standard manual morphometry. The prognostic ability of practicable manual morphometry is encouraging considering that this test was restricted to 12 nuclei using a stratified sampling strategy, whereas the gold standard manual method evaluated 100 nuclei with a complete sampling within random grid fields. Our findings (2-dimensional morphometry) stand in contrast to the conclusions of Casanova et al,^15^ who found limited prognostic value of the volume-weighted mean nuclear volume (3-dimensional morphometry). The benefit of algorithmic morphometry is that a large number of nuclei (usually >1000, complete sampling strategy) can be evaluated, increasing the representativeness of the measurement and thus increasing the prognostic value as compared to the manual measurements.

While most size measurements had a very similar prognostic relevance, the parameters 90th percentile/median (approximating the 2011 karyomegaly definition ^28^) were not particularly relevant in this study population due to the high correlation between the 90th percentile and median, which reduced the effect size of the quotient. Defining karyomegaly through the proportion of tumor cells with enlarged nuclei above a specific nuclear area (such as ≥38 µm^2^) seems to be more appropriate; however, it is impractical for evaluations using traditional light microscopy. Of note, the manual measurement of one of the largest nuclei in the image (maximum measurement by pathologists), reflecting the presence or absence of few karyomegalic cells, had surprisingly high prognostic value and may be a very practical prognostic test. Nuclear morphometry may represent a useful alternative to karyomegaly estimates for use in the 2011 grading system,^28^ providing that the laboratory uses digital microscopy. Validation of our findings is needed in larger study populations. Further studies are needed to evaluate the prognostic relevance of the different morphometric parameters of automated and practicable manual morphometry for further tumor types.

Another topic for future research is to combine different nuclear characteristics, such as nuclear size, shape, orientation, and spatial distribution.^1,32^ In our study, manual and algorithmic shape assessments had a markedly lower prognostic relevance than nuclear size parameters; however, it was beyond the scope of this study to evaluate whether nuclear shape assessments can add prognostic information when combined with nuclear size parameters.

Limitations of the present study are the lack of confirmation of the cause of death of the patients and the low number of cases with tumor-related death. These limitations justify a validation study on a second/independent outcome data set. The low number per outcome event also hindered multivariate analysis in determining whether a combination of several size and shape parameters had an added value.^55^ The prognostic results of the MC are as expected from the previous literature,^9,10,12,45^ suggesting that this outcome data set is representative. A great advantage of this outcome population is that all patients were exclusively treated by complete surgical removal, eliminating the bias of variable treatment strategies on patient survival.

Tumor heterogeneity is interesting regarding sampling strategies of tumor regions and understanding tumor biology. The use of a deep learning–based algorithm allowed us to analyze several ROIs and thus enabled intratumoral comparison of morphometric measurements in ccMCT for the first time. Although we observed variability of the morphometric measurements between the different tumor regions, the number of ROIs used for prognostic evaluation generally had a minor effect on the determined AUC values. Although analysis of a single ROI provided a satisfactory prognostic interpretation of the case, a higher number of ROIs or potentially larger ROI sizes might be slightly beneficial, which should be evaluated in a future study in more detail. Of note, the maximum value of the analyzed ROI per case did not result in a higher discriminative ability of patient survival than the average of all ROIs. However, as we had selected the representative tumor regions without attention to nuclear features and restricted the analysis to a few ROIs, it remains unknown whether areas with the highest morphometric values in the tumor section would be favorable for prognostic assessment of the case.

It is intriguing that intratumoral heterogeneity of morphometry itself had a moderate to good discriminative ability for patient survival, even though the heterogeneity measurement was restricted to 5 tumor regions. It would be interesting to explore the distribution of nuclear parameters throughout the entire tumor sections, as has been previously done for nuclear morphometry in human tumors ^1^ or for the MC in ccMCT.^7,8^ Particularly, the prognostic test “proportion of hotspot ROI” would probably benefit from a more comprehensive analysis of the entire tumor section. These fully automated nuclear morphometry algorithms have great potential for employing intratumoral heterogeneity as a prognostic test.

## Conclusion

Poor rater reproducibility of anisokaryosis estimates hinders meaningful application of this test for routine tumor prognostication. An alternative to estimation is nuclear morphometry, the advantages of which include high reproducibility and the capability of determining meaningful prognostic thresholds based on the association with patient outcome. Although manual measurements of a larger number of cells are impractical for routine application, we have shown that assessment of a few (12) tumor nuclei using a stratified sampling strategy provides improved rater reproducibility (as compared with estimates) and a meaningful prognostic information (as compared with the gold standard manual morphometry method). In addition, we propose a deep learning–based algorithm that is capable of analyzing thousands of cells within seconds at low-computational costs. This study demonstrated a high-measurement accuracy and high prognostic value of fully automated nuclear morphometry for ccMCT. In this study population, morphometric parameters evaluating the nuclear area (such as the standard deviation or 90th percentile) were particularly prognostically relevant. The results of this study encourage the application of automated nuclear morphometry for routine tumor evaluation in laboratories with established digital workflows. A more thorough investigation of tumor heterogeneity and its prognostic value is warranted based on our preliminary findings with a relatively low number of tumor regions.

## Supplemental Material

sj-pdf-1-vet-10.1177_03009858241295399 – Supplemental material for Nuclear pleomorphism in canine cutaneous mast cell tumors: Comparison of reproducibility and prognostic relevance between estimates, manual morphometry, and algorithmic morphometry Supplemental material, sj-pdf-1-vet-10.1177_03009858241295399 for Nuclear pleomorphism in canine cutaneous mast cell tumors: Comparison of reproducibility and prognostic relevance between estimates, manual morphometry, and algorithmic morphometry by Andreas Haghofer, Eda Parlak, Alexander Bartel, Taryn A. Donovan, Charles-Antoine Assenmacher, Pompei Bolfa, Michael J. Dark, Andrea Fuchs-Baumgartinger, Andrea Klang, Kathrin Jäger, Robert Klopfleisch, Sophie Merz, Barbara Richter, F. Yvonne Schulman, Hannah Janout, Jonathan Ganz, Josef Scharinger, Marc Aubreville, Stephan M. Winkler, Matti Kiupel and Christof A. Bertram in Veterinary Pathology
