# Supplementary material for: Nuclear pleomorphism in canine cutaneous mast cell tumors: Comparison of reproducibility and prognostic relevance between estimates, manual morphometry, and algorithmic morphometry
Source: Vet Pathol. 2024 Nov 19;62(2):161–77. doi: 10.1177/03009858241295399 (PMC11874577; doi:10.1177/03009858241295399)
Supplement: sj-pdf-1-vet-10.1177_03009858241295399 – Supplemental material for Nuclear pleomorphism in canine cutaneous mast cell tumors: Comparison of reproducibility and prognostic relevance between estimates, manual morphometry, and algorithmic morphometry [file sj-pdf-1-vet-10.1177_03009858241295399.pdf]

Supplemental Materials

Nuclear pleomorphism in canine cutaneous mast cell tumors: Comparison of reproducibility and prognostic relevance between estimates, manual morphometry, and algorithmic morphometry

Andreas Haghofer, Eda Parlak, Alexander Bartel, Taryn A. Donovan, Charles-Antoine Assenmacher, Pompei Bolfa, Michael J. Dark, Andrea Fuchs-Baumgartinger, Andrea Klang, Kathrin Jäger, Robert Klopfleisch, Sophie Merz, Barbara Richter, F. Yvonne Schulman, Hannah Janout, Jonathan Ganz, Josef Scharinger, Marc Aubreville, Stephan M. Winkler, Matti Kiupel, Christof A. Bertram

Addendum to the Study Methods

Gold Standard Manual Nuclear Morphometry (≥100 nuclei)

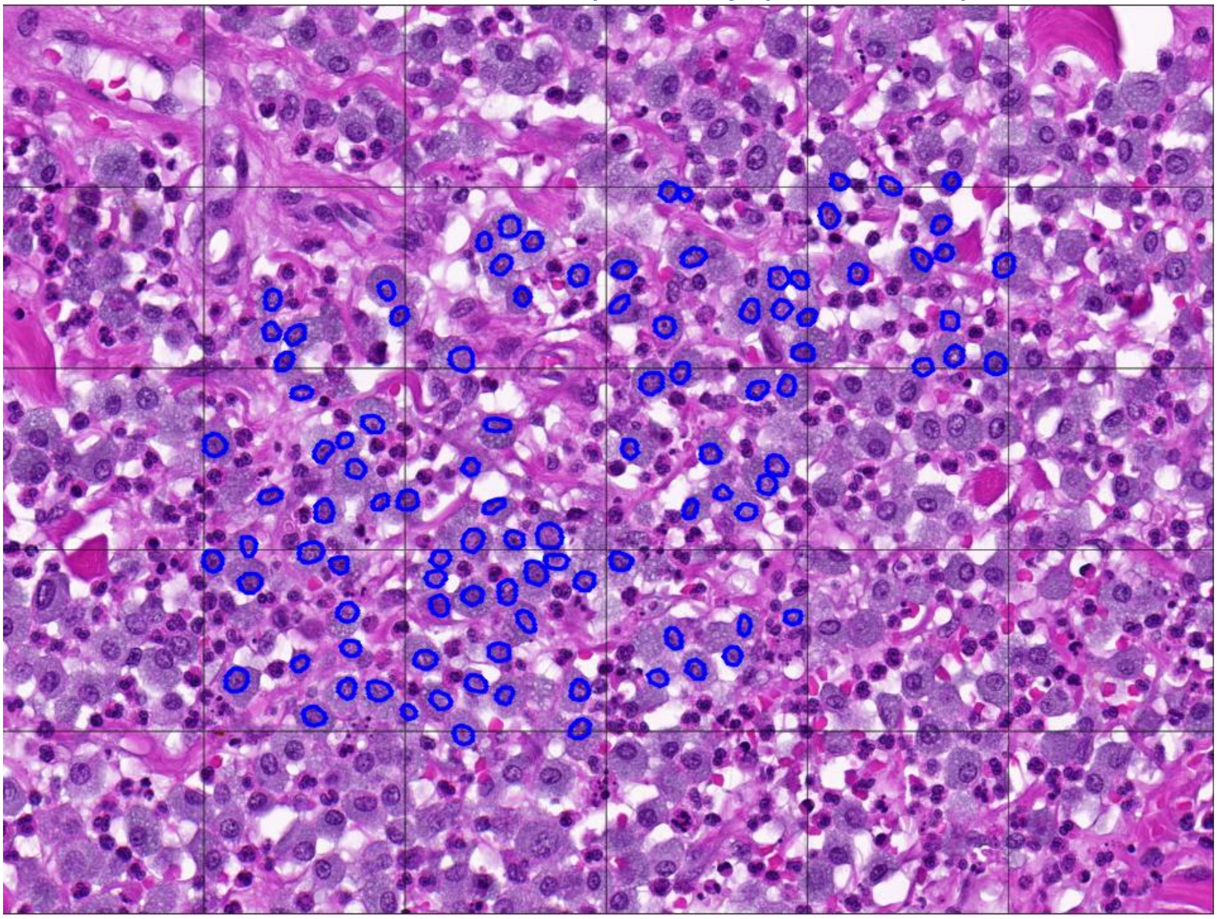

Supplemental Figure S1. Example image for the annotations of the gold standard manual nuclear morphometry with ≥100 nuclei (in this case 102 nuclei). A 5x6 grid (thin black lines) was overlayed on the image and the central grid fields were annotated in a meandering pattern. In this case 10 grid fields were completely annotated for tumor nuclei to reach the ≥100 nuclei.

Development of the Deep Learning-based Segmentation Model

Using the PyTorch lightning framework,[1] we developed a Unet ++ [4] -based segmentation model with RegNetY120 backbone [3] of the segmentation models package [2] to create binary masks where pixels corresponding to the nuclei of the initial slide image are depicted as positive foreground (ones) in front of a negative background (zeros). We trained our model for 2,000 epochs using the training and validation subsets of the ground truth dataset. During each epoch, all 61 training samples were split into batches of eight samples. Processing one batch per step, we trained in 7.625 steps, resulting in seven full batches and one batch with only five samples. The final model was selected as the best out of all processed epochs based on the validation loss (focal loss, evaluated on the validation subset). We applied data augmentation including extension techniques to the training data to regularize the training process (for the validation data, we only applied data extension without augmentation). The data augmentation methods used consisted of color and contrast adjustments combined with various distortion methods like elastic-, optical-, grid-distortion, and methods like shifting, scaling, and rotation to increase the variation in the available images to decrease overfitting and the impact of domain shift due to different imaging setups.

To extend our dataset, we multiplied the number of steps per epoch by a factor of 10, resulting in ~77 training steps. Using the same calculation, we also got 14 validation steps at the end of each epoch. In order to generate the necessary images for the extended number of batches, we used image cropping. This involves randomly selecting image regions of 512x512 pixels out of the original dataset, with images of 1601x1201 pixels, until the required number of crops was reached. Exclusively for the training steps, we resampled new crops for every epoch and applied data augmentation to each crop.

After applying the deep learning model for segmentation of individual nuclei, we used a filter mechanism as post-processing for the exclusion of objects that did not represent a valid nucleus. We excluded all segmented objects with an area below approximately 7 µm² within our segmentation mask, thus we have removed nuclei that are too small and do not represent complete (oversegmentation) or valid mast cell nuclei (such as eosinophils etc.). The filter threshold of approximately 7 µm² was decided based on the size distribution of the mast cell nuclei annotations of the training subset of the ground truth dataset and the appropriateness of this filter threshold was verified on the test subset of the ground truth dataset, as shown in Supplemental Figure S2.

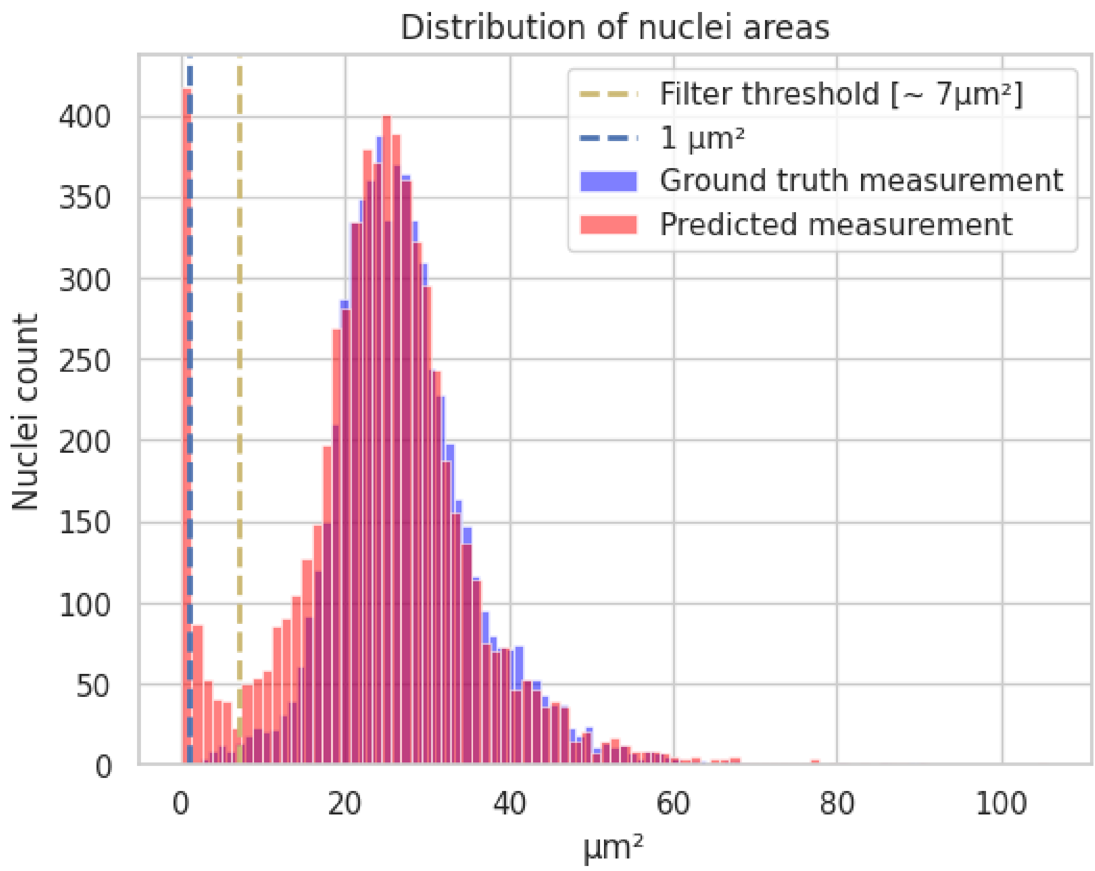

Supplemental Figure S2. Frequency distribution of nuclear size of the ground truth annotations and predictions of the deep learning-based segmentation model on the test subset of the ground truth dataset.

References:

1. Falcon W: PyTorch Lightning. GitHub. Note: https://github.com/PyTorchLightning/pytorch-lightning,. 2019
2. Iakubovskii P. Segmentation Models Pytorch. Note: https://github.com/qubvel/segmentation_models.pytorch. 2019.
3. Radosavovic I, Kosaraju RP, Girshick R, He K, Dollár P: Designing network design spaces. *In*: Proceedings of the IEEE/CVF conference on computer vision and pattern recognition, pp. 10428-10436. 2020
4. Zhou Z, Siddiquee MMR, Tajbakhsh N, Liang J. UNet++: A Nested U-Net Architecture for Medical Image Segmentation. *Deep Learn Med Image Anal Multimodal Learn Clin Decis Support (2018)*. 2018;11045**:** 3-11.

Rater reproducibility

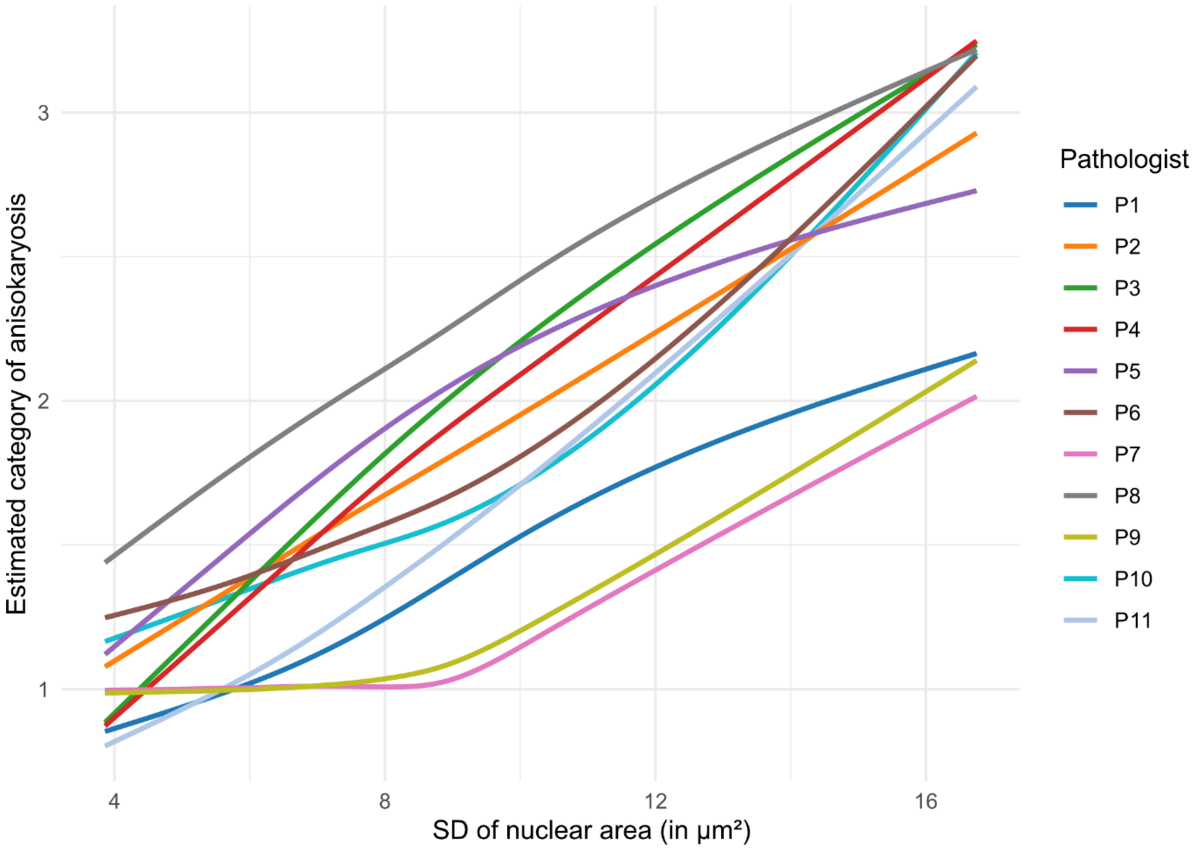

Supplemental Figure S3. Illustration of the increase in the average three-tier anisokaryosis estimate (time point 1, outcome dataset) depending on the algorithmic standard deviation (SD) of the nuclear area for each pathologist (curves determined by linear regression). The curves show which anisokaryosis category was likely assigned by the corresponding pathologist to a case depending on the SD of nuclear area measured. Curves were smoothed using a spline regression.

Method Accuracy

Pathologists’ Estimates

Supplemental Table S1. Frequency of estimated karyomegaly categories (two-tier) per case of the test subset of the ground truth dataset for the 9 pathologists (time point 2).

Rank of case (based on second column)	Algorithmic SD of nuclear area	Frequency of estimated karyomegaly categories	
		Absent	Present
1	5.15 µm²	9	0
2	5.73 µm²	5	4
3	5.79 µm²	7	2
4	5.82 µm²	8	1
5	5.88 µm²	8	1
6	6.03 µm²	9	0
7	6.47 µm²	0	9
8	6.54 µm²	8	1
9	7.50 µm²	8	1
10	7.56 µm²	6	3
11	10.24 µm²	7	2
12	10.92 µm²	0	9
13	12.58 µm²	0	9

SD, standard deviation

Supplemental Table S2. Frequency of estimated anisokaryosis categories (three-tier) per case of the test subset of the ground truth dataset for the 9 pathologists (time point 2).

Rank of case (based on second column)	Algorithmic SD of nuclear area	Frequency of estimated anisokaryosis categories		
		No – mild	Moderate	Severe
1	5.15 µm²	6	3	0
2	5.73 µm²	4	3	2
3	5.79 µm²	3	4	2
4	5.82 µm²	6	3	0
5	5.88 µm²	6	3	0
6	6.03 µm²	7	1	1
7	6.47 µm²	0	5	4
8	6.54 µm²	5	2	2
9	7.50 µm²	5	3	1
10	7.56 µm²	3	5	1
11	10.24 µm²	4	2	3
12	10.92 µm²	2	3	4
13	12.58 µm²	0	3	6

SD, standard deviation

Gold Standard Manual Nuclear Morphometry (≥100 nuclei)

Supplemental Table S3. Root mean squared error (RMSE) for the different morphometric parameters comparing the measurements of the gold standard manual annotations (≥100 nuclei) to the ground truth measurements for the 13 cases of the test dataset. It should be noted that the ground truth measurements include all neoplastic nuclei in the images while the manual annotations only include a subset (N ≥ 100) of these.

Feature	Morphometric parameter	Root mean squared error (RMSE)	Value range ground truth measurement	RMSE to range ratio (coefficient of variation)
Size (area in µm²)	Mean	2.35 µm²	22.59 – 38.43 µm²	14.8%
	Median	2.32 µm²	22.51 – 37.47 µm²	15.5%
	SD	0.84 µm²	5.15 – 12.58 µm²	11.3%
	90th percentile (P)	2.40 µm²	30.81 – 52.38 µm²	11.1%
	90th P / median	0.083	1.240 – 1.516	30.2%
	Mean of the largest 10%	2.97 µm²	34.18 – 63.86 µm²	10.0%
	Percentage large nuclei (>37.8 µm²)	4.31%	1.16 – 48.91%	9.0%
	Percentage large nuclei (>50.3 µm²)	1.86%	0 – 11.95%	15.6%
	Skewness	0.587	-0.583 – 1.617	26.7
Shape	Mean eccentricity	0.053	0.533 – 0.676	37.2%
	SD of eccentricity	0.011	0.128 – 0.149	55.9%
	Skewness eccentricity	0.320	-1.513 – -0.042	21.7%
	Mean solidity	0.004	0.943 – 0.960	23.3%
	SD of solidity	0.017	0.009 – 0.055	36.6%
	Percentage of nuclei with indentation (solidity <0.913)	2.32%	0.19 - 7.55%	31.5%
	Skewness solidity	4.52	-9.24 – -1.30	57.0%

SD, standard deviation

Practicable Manual Nuclear Morphometry (12 nuclei)

Supplemental Table S4. Root mean squared error (RMSE) for the standard deviation of nuclear area of the practicable manual nuclear morphometry (12 nuclei) compared to the ground truth measurements (standard deviation of the area) for the 13 cases of the test dataset and each individual pathologist.

Pathologist	Root mean squared error (RMSE)	Value range ground truth measurement	RMSE to range ratio (coefficient of variation)
Pathologist 1	3.79 µm²	5.15 – 12.58 µm²	51.0%
Pathologist 2	5.32 µm²	5.15 – 12.58 µm²	71.7%
Pathologist 3	3.93 µm²	5.15 – 12.58 µm²	52.8%
Pathologist 5	4.41 µm²	5.15 – 12.58 µm²	59.4%
Pathologist 6	7.53 µm²	5.15 – 12.58 µm²	101.3%
Pathologist 7	4.83 µm²	5.15 – 12.58 µm²	65.1%
Pathologist 8	4.05 µm²	5.15 – 12.58 µm²	54.5%
Pathologist 10	3.97 µm²	5.15 – 12.58 µm²	53.5%
Pathologist 11	5.65 µm²	5.15 – 12.58 µm²	76.0%
Mean measurement	4.18 µm²	5.15 – 12.58 µm²	56.3%

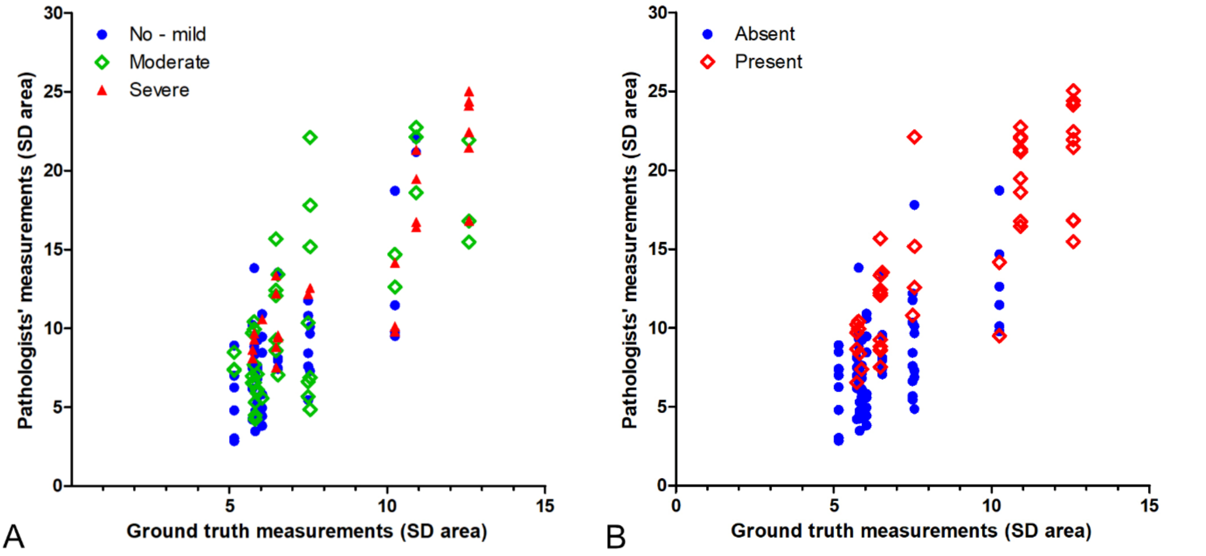

Supplemental Figure S4. Scatter plot of the standard deviation (SD) of nuclear area comparing the pathologists' (N = 9) measurements with the ground truth measurements for the 13 cases of the test subset of the ground truth dataset. **A)** Each datapoint is classified by the estimated degree of three-tier anisokaryosis assigned by the same pathologist. **B)** Each datapoint is classified by the estimated degree of two-tier karyomegaly assigned by the same pathologist.

Supplemental Table S5. Root mean squared error (RMSE) for the <u>mean of nuclear area</u> of the <u>practicable manual nuclear morphometry (12 nuclei)</u> compared to the ground truth measurements (standard deviation of the area) for the 13 cases of the test dataset and each individual pathologist.

**Pathologist**	**Root mean squared error (RMSE)**	**Value range ground truth measurement**	**RMSE to range ratio (coefficient of variation)**
Pathologist 1	4.74	22.59 – 38.43 µm²	29.9%
Pathologist 2	8.04	22.59 – 38.43 µm²	50.8%
Pathologist 3	4.67	22.59 – 38.43 µm²	29,5%
Pathologist 5	5.33	22.59 – 38.43 µm²	33.7%
Pathologist 6	4.16	22.59 – 38.43 µm²	26.2%
Pathologist 7	9.77	22.59 – 38.43 µm²	61.7%
Pathologist 8	5.01	22.59 – 38.43 µm²	31.6%
Pathologist 10	4.05	22.59 – 38.43 µm²	25.6%
Pathologist 11	12.40	22.59 – 38.43 µm²	78.3%
Mean measurements	5.38	22.59 – 38.43 µm²	34.0%

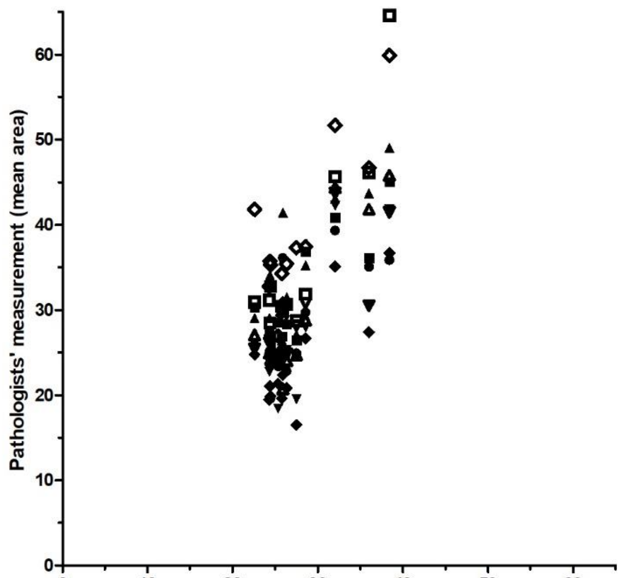

Supplemental Figure S5. Scatter plot of the mean nuclear area comparing the pathologists' (N = 9) measurements with the ground truth measurements for the 13 cases of the test subset of the ground truth dataset. The different symbols represent the different pathologists.

Supplemental Table S6. Root mean squared error (RMSE) for the different morphometric parameters comparing the measurements of the deep learning-based algorithm to the ground truth measurements for the 13 cases of the test dataset.

**Feature**	**Morphometric parameter**	**Root mean squared error (RMSE)**	**Value range ground truth measurement**	**RMSE to range ratio (coefficient of variation)**
Size (area in µm²)	Mean	2.78 µm²	22.59 – 38.43 µm²	17.6%
	Median	2.54 µm²	22.51 – 37.47 µm²	17.0%
	SD	1.69 µm²	5.15 – 12.58 µm²	22.8%
	90th percentile (P)	3.04 µm²	30.81 – 52.38 µm²	14.1%
	90th P / median	0.081	1.240 – 1.516	29.4%
	Mean of the largest 10%	4.83 µm²	34.18 – 63.86 µm²	16.3%
	Percentage large nuclei (>37.8 µm²)	6.17%	1.16 – 48.91%	12.9%
	Percentage large nuclei (>50.3 µm²)	1.37%	0 – 11.95%	11.5%
	Skewness	0.802	-0.583 – 1.617	36.4%
Shape	Mean eccentricity	0.022	0.533 – 0.676	15.5%
	SD of eccentricity	0.015	0.128 – 0.149	71.8%
	Skewness eccentricity	0.285	-1.513 – -0.042	19.4%
	Mean solidity	0.003	0.943 – 0.960	16.7%
	SD of solidity	0.013	0.009 – 0.055	28.5%
	Percentage of nuclei with indentation (solidity <0.913)	3.54%	0.19 - 7.55%	48.2%
	Skewness solidity	3.69	-9.24 – -1.30	46.5%

SD, standard deviation

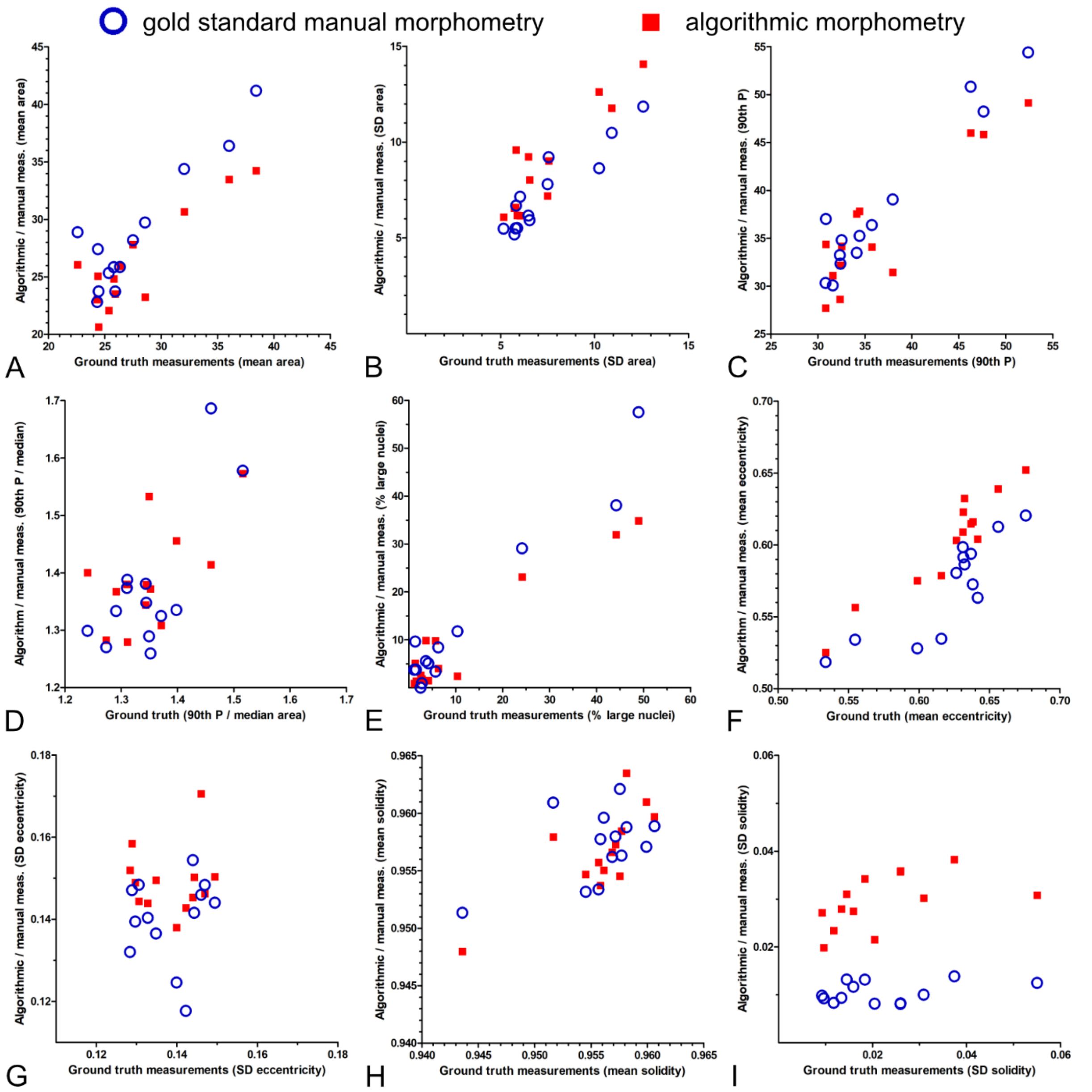

Supplemental Figure S6. Scatter plot of the different morphometric parameters comparing the algorithmic measurements (red squares, y-axis) and the gold standard manual measurements of ≧100 nuclei (blue circles, y-axis) with the ground truth measurements (x-axis) for the 13 cases of the test dataset. **A)** mean nuclear area in µm², **B)** standard deviation (SD) of nuclear area in µm², **C)** 90th percentile (90thP) of nuclear area in µm², **D)** 90thP / median nuclear area, **E)** percentage (%) of large nuclei with an area >37.8 µm², **F)** mean eccentricity **G)** SD of eccentricity, **H)** mean solidity, **I)** SD of solidity

Meas., measurments

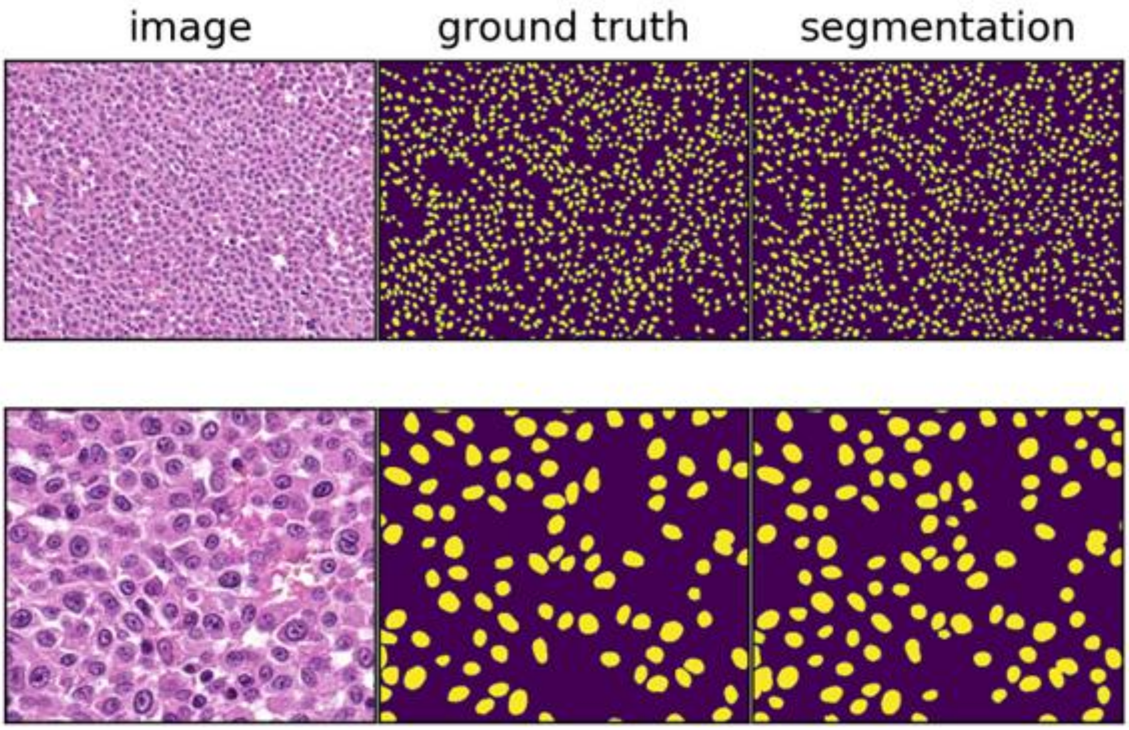

Supplemental Figure S7. Comparison of the ground truth and the segmentation mask derived from the deep learning-based model from an image (complete image on the top and smaller image region of the same image on the bottom) of the test subset of the ground truth dataset. The yellow areas represent the segmented mast cell tumor nuclei in the binary mask.

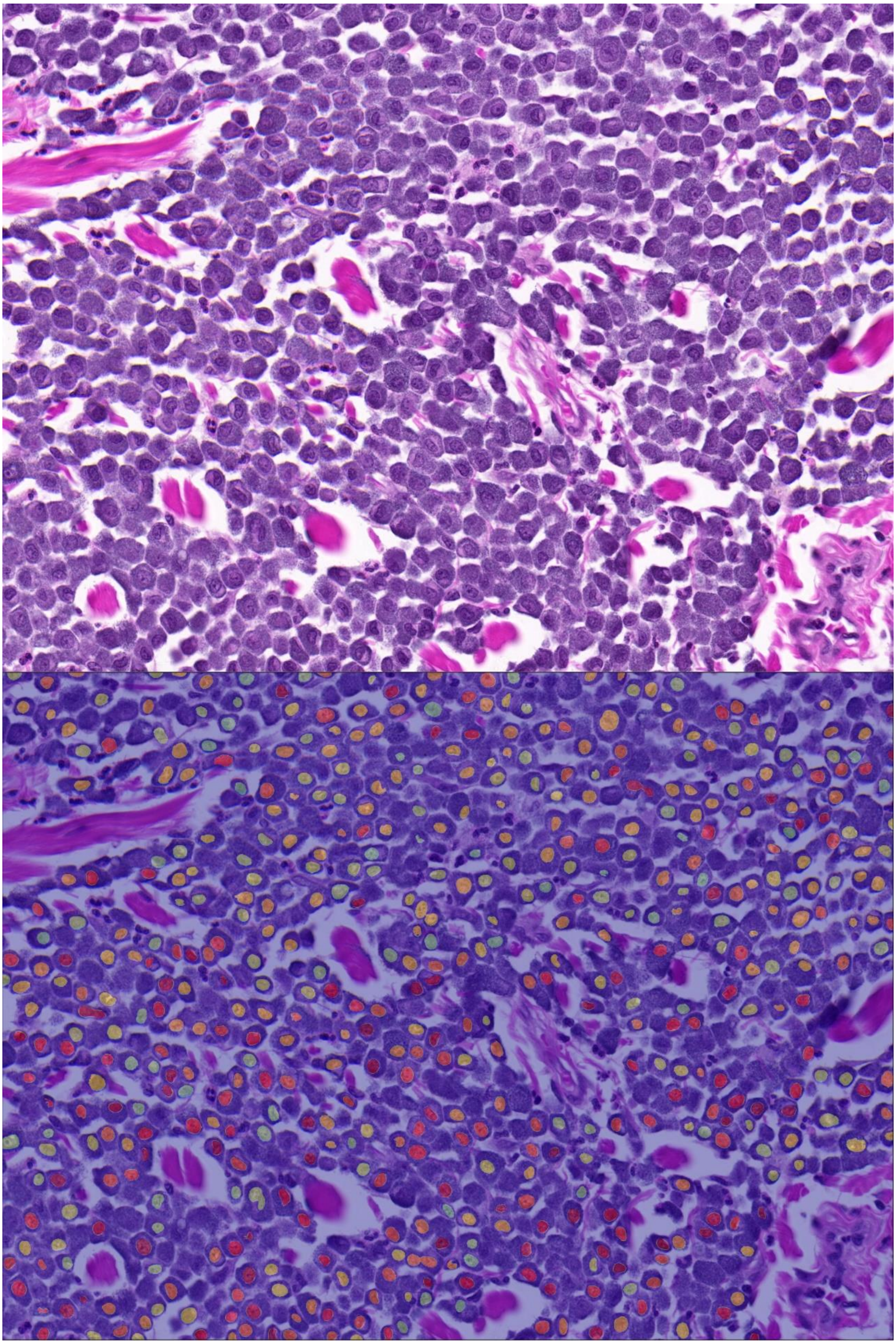

Supplemental Figure S8. Example of an image from the outcome dataset with numerous intracytoplasmic metachromatic granules partially obscuring the nuclei of the neoplastic mast cells (upper image). The deep learning-based algorithm was able to appropriately segment most neoplastic nuclei (lower image).

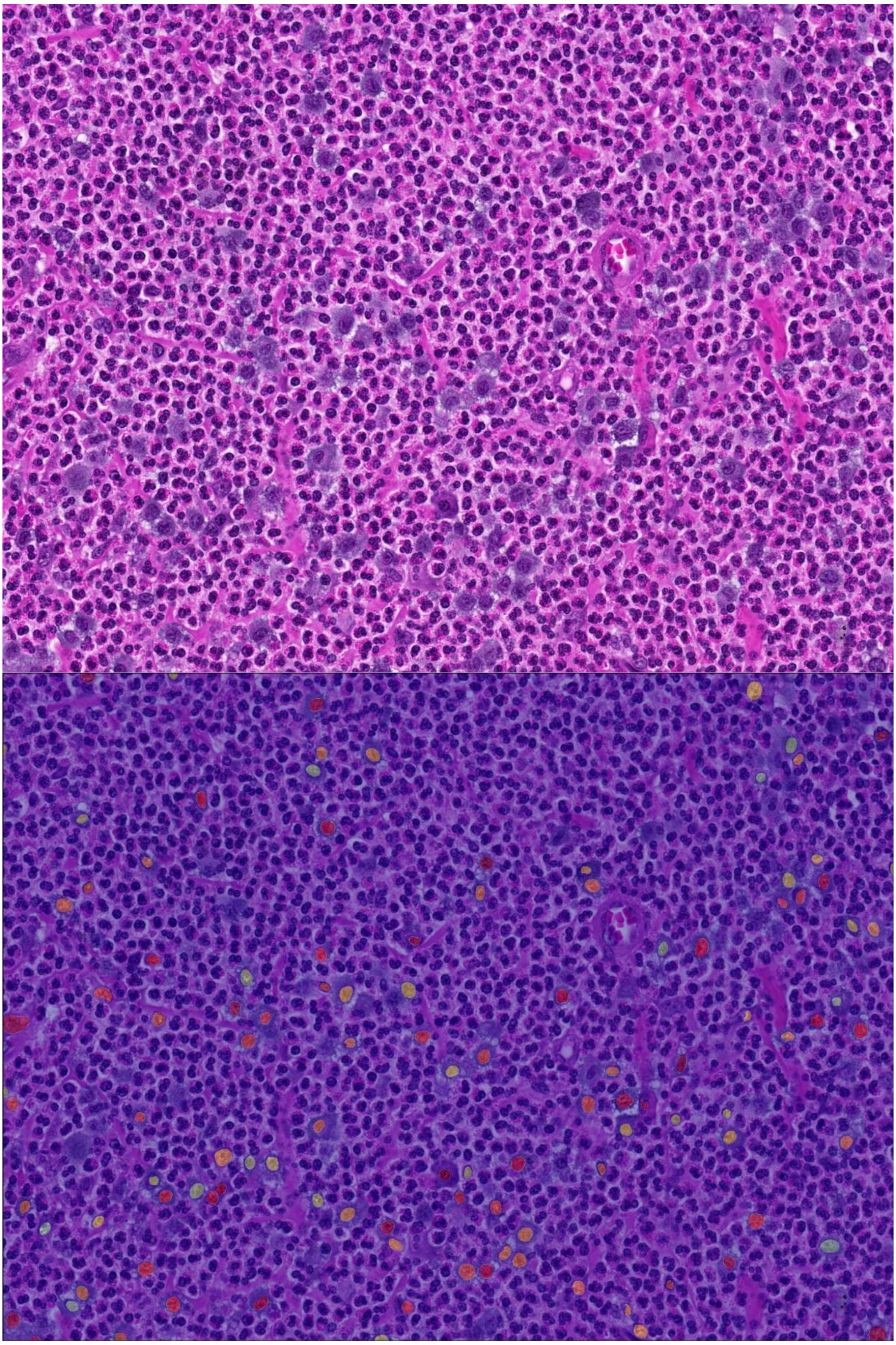

Supplemental Figure S9. Example of an image from the outcome dataset with numerous eosinophils between the neoplastic mast cells (upper image). The deep learning-based algorithm falsely segmented very few eosinophils.

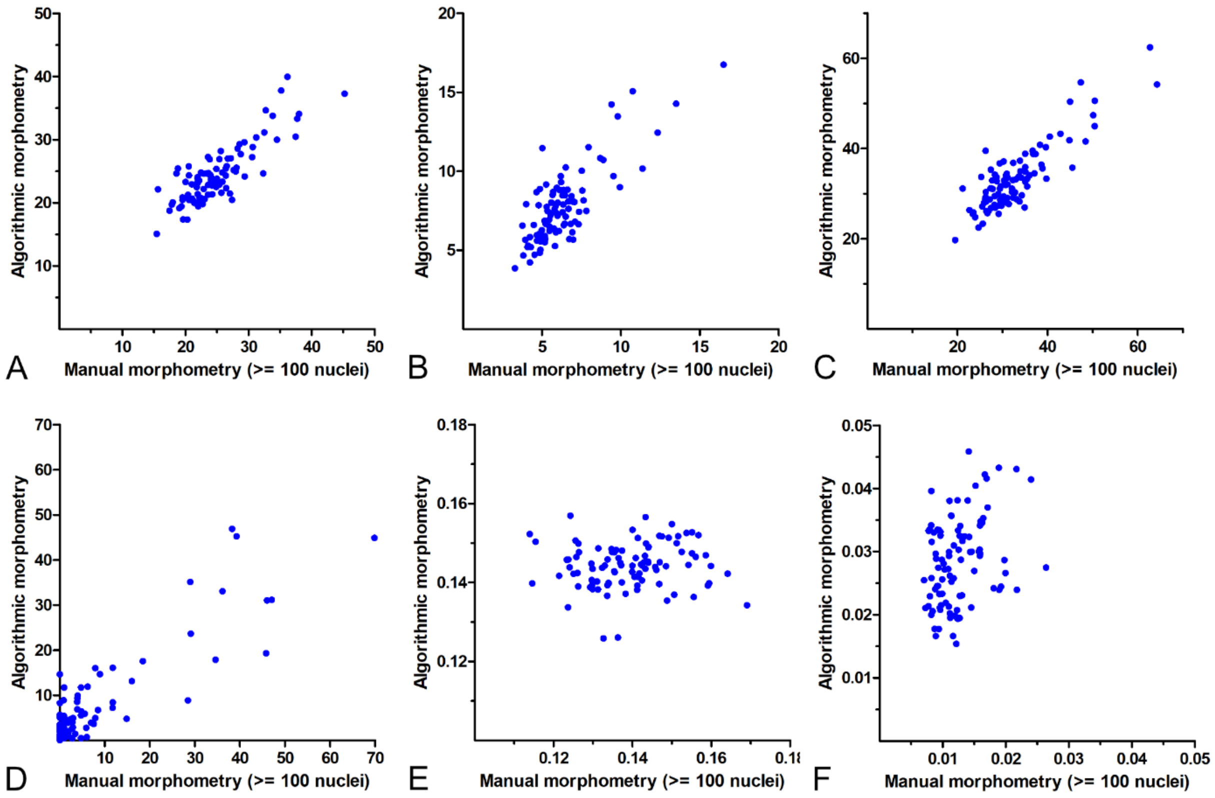

Supplemental Figure S10. Scatter plot comparing measurements of the gold standard manual morphometry (≥100 nuclei) and algorithmic morphometry for the 96 outcome cases. **A)** mean nuclear area in μm^2 (r = 0.844), **B)** standard deviation (SD) of nuclear area in μm^2 (r = 0.805), **C)** 90th percentile (90thP) of nuclear area in μm^2 (r = 0.66), **D)** percentage (%) of large nuclei with an area >37.8 μm^2 (r = 0.880), **E)** SD of eccentricity (r = 0.062), **F)** SD of solidity (r = 329).

r, Pearson correlation coefficients

Correlation of Morphometric Parameters

Supplemental Table S7. Correlation coefficients (Pearson method) for comparison of the individual area parameters measured by gold standard manual nuclear morphometry (≥100 nuclei) in the 96 outcome cases. The cells in the table are color coded with correlation coefficients of 0 being white and 1 being dark blue.

Area parameters	Mean	Median	SD	90th P	90th P / median	Mean largest 10%	% above 37.8 µm²	% above 50.3 µm²
Mean	1							
Median	0.991	1						
SD	0.850	0.800	1					
90th percentile (P)	0.966	0.936	0.941	1				
90th P / median	0.223	0.116	0.629	0.455	1			
Mean of the largest 10%	0.957	0.922	0.960	0.987	0.452	1		
Percent of cells above 37.8 µm²	0.901	0.881	0.870	0.911	0.332	0.924	1	
Percent of cells above 50.3 µm²	0.731	0.692	0.813	0.802	0.462	0.805	0.867	1

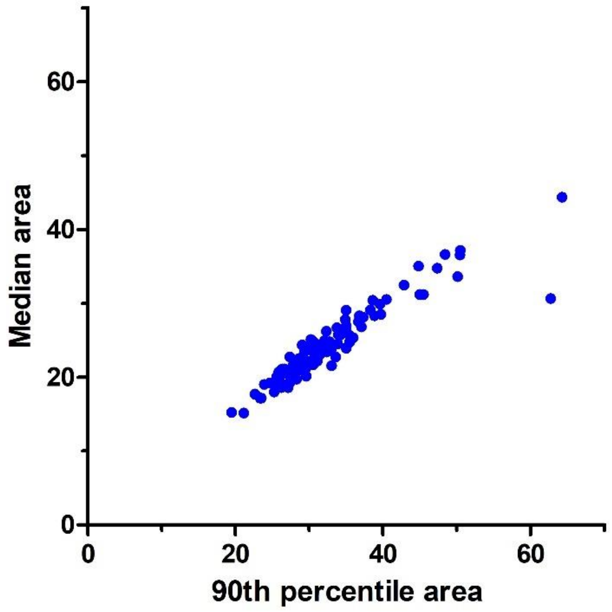

Supplemental Figure S11. Scatter plot comparing the 90th percentile and median nuclear area measurements (in µm²) of the 96 outcome cases measured by gold standard manual nuclear morphometry (≥100 nuclei). These two morphometric parameters have a Pearson correlation coefficient of 0.936.

Supplemental Table S8. Correlation coefficients (Pearson method) for comparison of the individual area parameters measured by algorithmic nuclear morphometry in the 96 outcome cases. The cells in the table are color coded with correlation coefficients of 0 being white and 1 being dark blue.

Area parameters	Mean	Median	SD	90th P	90th P / median	Mean largest 10%	% above 37.8 µm²	% above 50.3 µm²
Mean	1							
Median	0.996	1						
SD	0.936	0.910	1					
90th percentile (P)	0.985	0.970	0.975	1				
90th P / median	0.508	0.446	0.750	0.640	1			
Mean of the largest 10%	0.974	0.955	0.988	0.943	0.665	1		
Percent of cells above 37.8 µm²	0.930	0.911	0.925	0.948	0.606	0.943	1	
Percent of cells above 50.3 µm²	0.811	0.776	0.850	0.860	0.642	0.848	0.912	1

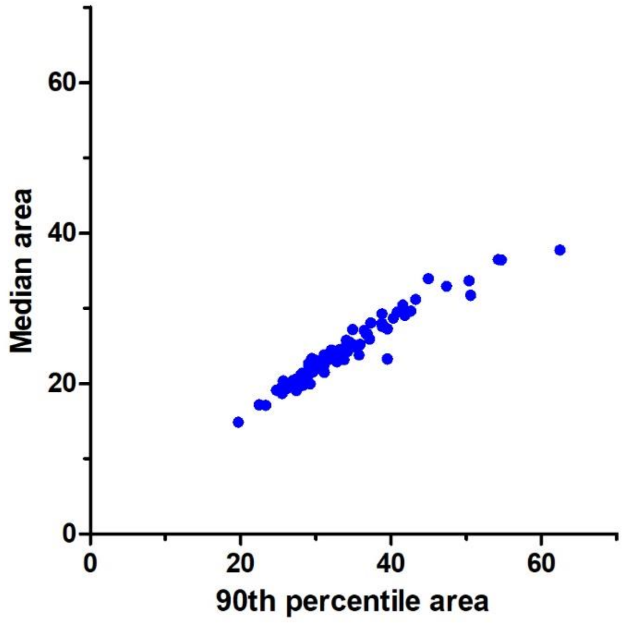

Supplemental Figure S12. Scatter plot comparing the 90th percentile and median nuclear area measurements (in µm²) of the 96 outcome cases measured by algorithmic nuclear morphometry. These two morphometric parameters have a Pearson correlation coefficient of 0.970.

Prognostic Value

Demographic information of the study population

Supplemental Table S9. Summary of the demographic information of the study population of the outcome dataset.

Demographic information	Case number
Breed	mixed-breed (N = 21), Labrador Retriever (N = 21), Boxer (N O 11), Golden Retriever (N = 10), Pug (N = 4), Basset Hound (N = 4), Boston Terrier (N = 3), Cocker Spaniel (N = 3), and other breeds (N = 19)
Sex	spayed female (N = 51), castrated males (N = 33), intact females (N = 7) and intact males (N = 5)
Age	older than 10 years (N = 26), between 6 – 9 years (N = 40), between 3 – 5 years (N = 27), and younger than 2 years (N = 3)

The outcome dataset comprised of 96 canine cutaneous mast cell tumors (one tumor per patient) each with confirmed dermal location of the tumor (with possible subcutaneous infiltration). Mast cell tumors with exclusive subcutaneous location or mucocutaneous location were excluded. No dog received any therapy for the mast cell tumor besides excisional surgery with curative intent. Routine margin evaluation of the tumor during diagnostic service confirmed complete surgical removal.

Frequency distribution

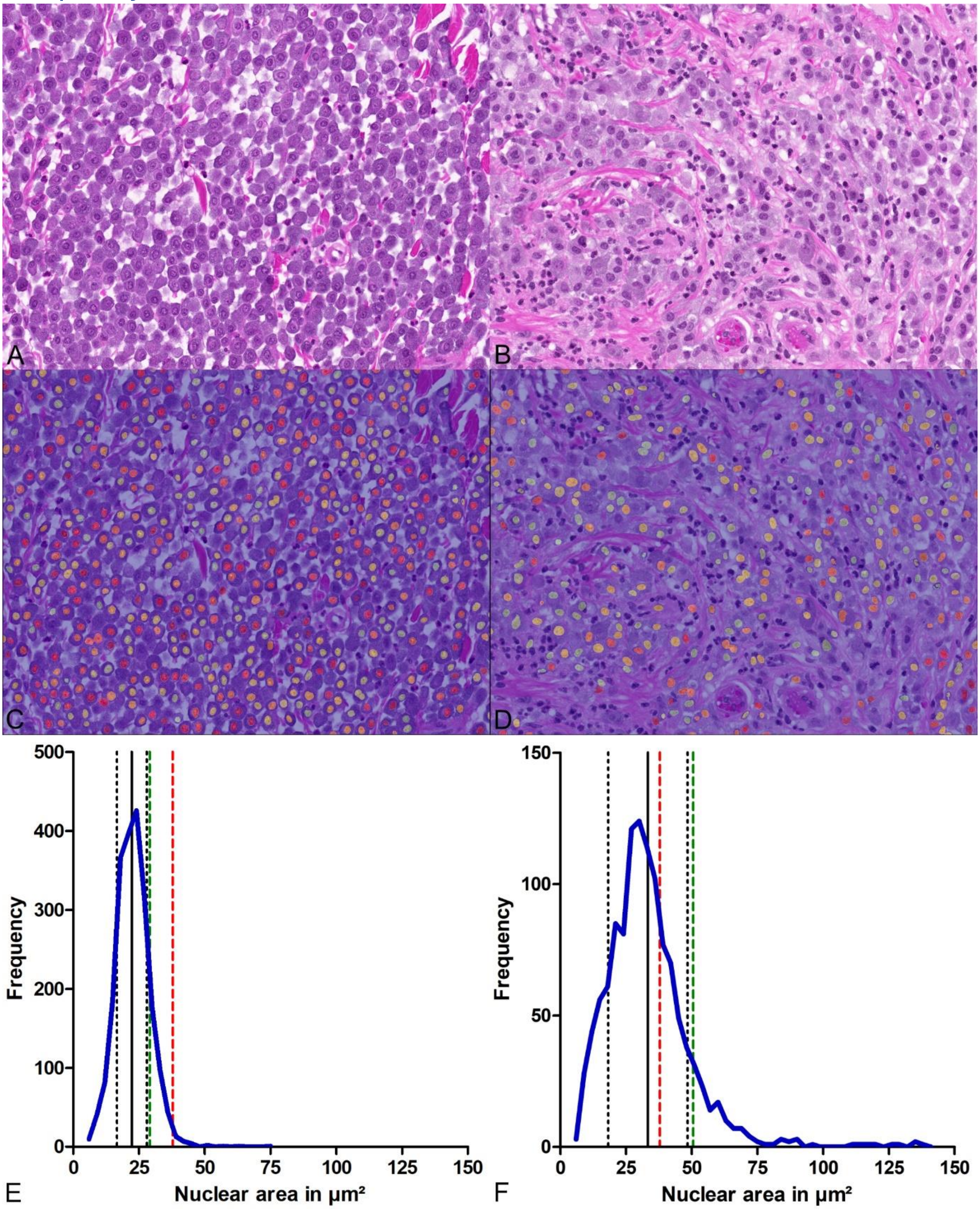

Supplemental Figure S13. (A, B) Histological images, (C, D) segmentation maps of the deep learning-based model (middle images), and (E, F) frequency distributions of nuclear area measurements for a canine cutaneous mast cell tumor without tumor-related mortality (A, C, E) and a tumor with tumor-related mortality (B, D, F). A, B) Five of these images (size: 0.1185 mm²) were available per tumor case for algorithmic morphometry. C) Algorithmic segmentation of these five images of this tumor resulted in 2170 objects. D) Algorithmic segmentation of five images of this tumor resulted in 1188 objects. E and F) The algorithmically predicted objects (see

images C and D) were used for analysis of the frequency distribution. The bin width for the frequency distribution curves (blue lines) are 3 µm². E) The nuclear size of this indolent tumor has a mean of 22.34 µm² (black line), a standard deviation of 5.68 µm² (black dotted lines are the mean ± the standard deviation), a 90th percentile of 29.12 µm² (green dotted line) and the percentage of large nuclei (>37.8 µm², red dotted line) is 1.12%. F) The nuclear size of this aggressive tumor has a mean of 33.32 µm² (black line), a standard deviation of 15.07 µm² (black dotted lines are the mean ± the standard deviation), a 90th percentile of 50.57 µm² (green dotted line) and the percentage of large nuclei (>37.8 µm², red dotted line) is 31.18%. Figure D shows few connected nuclei (undersegmentation), which explains the few extremely large objects (>100 µm²) in the frequency distribution.

Area Under the Curve

Gold Standard Manual Nuclear Morphometry (≥100 nuclei)

Supplemental Table S10. Area under the ROC curve (AUC) with 95% confidence intervals (95% CI) for the individual parameters of gold standard manual nuclear morphometry (≥100 nuclei) regarding tumor-specific survival throughout the entire follow-up period. The mitotic count (benchmark) by the first pathologist archived an AUC of 0.885 (95%CI: 0.765 - 1.00, $p < 0.001$).

**Morphometric feature**	**Morphometric parameter**	**AUC (95% CI)**
Size (area in µm²)	Mean	0.853 (0.709 – 0.997) **
	Median	0.848 (0.707 – 0.989) **
	Standard deviation (SD)	0.839 (0.701 – 0.977) **
	90th percentile (90th P)	0.861 (0.725 – 0.997) **
	90th P/ median	0.710 (0.587 – 0.832) *
	Mean of the largest 10% of the nuclei	0.856 (0.720 – 0.993) **
	Percentage of large nuclei (>37.8 µm²)	0.837 (0.675 – 0.999) **
	Percentage of large nuclei (>50.3 µm²)	0.804 (0.642 – 0.966) **
	Skewness	0.513 (0.364 – 0.661)
Shape	Mean eccentricity	0.557 (0.416 – 0.698)
	SD of eccentricity	0.602 (0.414 – 0.791)
	Skewness eccentricity	0.526 (0.391 – 0.662)
	1 - Mean solidity	0.348 (0.190 – 0.505)
	SD of solidity	0.754 (0.595 – 0.913) *
	Percentage of nuclei with indentation (solidity <0.913)	0.723 (0.578 – 0.867) *
	Percentage of nuclei with indentation (solidity <0.936)	0.582 (0.434 – 0.730)
	Percentage of nuclei with indentation (solidity < 0.943)	0.436 (0.293 – 0.579)

* $p \leq 0.05$; ** $p \leq 0.001$

Supplemental Table S11. Area under the ROC curve (AUC) with 95% confidence intervals (95% CI) for the individual parameters of gold standard manual nuclear morphometry (≥100 nuclei) regarding tumor-specific survival at 12 months after surgery. Cases that had died within the first 12 months after surgery due to an ccMCT-unrelated cause (N = 6) were excluded for this analysis. The mitotic count (benchmark) by the first pathologist archived an AUC of 0.890 (95% CI: 0.797 – 0.982; p < 0.001).

**Morphometric feature**	**Morphometric parameter**	**AUC (95% CI)**
Size (area in µm²)	Mean	0.795 (0.618 – 0.972) **
	Median	0.795 (0.623 – 0.967) **
	Standard deviation (SD)	0.806 (0.643 – 0.968) **
	90th percentile (90th P)	0.811 (0.646 – 0.976) **
	90th P/ median	0.704 (0.575 – 0.834) *
	Mean of the largest 10% of the nuclei	0.803 (0.636 – 0.970) **
	Percentage of large nuclei (>37.8 µm²)	0.775 (0.580 – 0.970) *
	Percentage of large nuclei (>50.3 µm²)	0.780 (0.590 – 0.970) *
	Skewness	0.481 (0.323 – 0.639)
Shape	Mean eccentricity	0.575 (0.427 – 0.724)
	SD of eccentricity	0. 595 (0.369 – 0.821)
	Skewness eccentricity	0.503 (0.349 – 0.657)
	1 - Mean solidity	0.358 (0.184 – 0.532)
	SD of solidity	0.789 (0.650 – 0.928) **
	Percentage of nuclei with indentation (solidity <0.913)	0.711 (0.570 – 0.853) *

* p ≤ 0.05; ** p ≤ 0.001

Supplemental Table S12. Area under the ROC curve (AUC) with 95% confidence intervals (95% CI) for the individual parameters of gold standard manual nuclear morphometry (≥100 nuclei) regarding overall mortality at 12 months after surgery (cases with death within the first 12 months after surgery regardless of cause compared to cases that survived for at least 12 months). The mitotic count (benchmark) by the first pathologist archived an AUC of 0.744 (95% CI: 0.596 – 0.891, p = 0.001).

**Morphometric feature**	**Morphometric parameter**	**AUC (95% CI)**
Size (area in µm²)	Mean	0.705 (0.530 – 0.879) *
	Median	0.707 (0.536 – 0.879) *
	Standard deviation (SD)	0.683 (0.513 – 0.852) *
	90th percentile (90th P)	0.705 (0.534 – 0.875) *
	90th P / median	0.652 (0.511 – 0.793) *
	Mean of the largest 10% of the nuclei	0.695 (0.523 – 0.866) *
	Percentage of large nuclei (>37.8 µm²)	0.665 (0.475 – 0.854)
	Percentage of large nuclei (>50.3 µm²)	0.700 (0.530 – 0.870) *
	Skewness	0.516 (0.371 – 0.660)
Shape	Mean eccentricity	0.542 (0.397 – 0.687)
	SD of eccentricity	0.519 (0.331 – 0.707)
	Skewness eccentricity	0.459 (0.323 – 0.594)
	1 - Mean solidity	0.381 (0.228 – 0.538)
	SD of solidity	0.627 (0.445 – 0.810)
	Percentage of nuclei with indentation (solidity <0.913)	0.610 (0.451 – 0.769)

* p ≤ 0.05; ** p ≤ 0.001

Supplemental Table S13. Area under the ROC curve (AUC) with 95% confidence intervals (95% CI) for practicable manual nuclear morphometry (12 nuclei) of the individual pathologists regarding tumor-specific survival throughout the entire follow-up period.

**Path-ologist**	**AUC (95% CI)**		
	**Mean area**	**SD of area**	**Maximum area**
P1	0.957 (0.917 – 0.998)	0.905 (0.842 – 0.969)	0.925 (0.870 – 0.980)
P2	0.898 (0.820 – 0.976)	0.852 (0.754 – 0.949)	0.898 (0.828 – 0.967)
P3	0.885 (0.771 – 0.999)	0.890 (0.781 – 0.999)	0.886 (0.755 – 1.00)
P5	0.883 (0.791 – 0.976)	0.823 (0.678 – 0.968)	0.841 (0.717 – 0.964)
P6	0.949 (0.902 – 0.997)	0.835 (0.741 – 0.929)	0.880 (0.806 – 0.953)
P7	0.912 (0.834 – 0.990)	0.960 (0.918 – 1.00)	0.956 (0.908 – 1.00)
P8	0.862 (0.747 – 0.979)	0.842 (0.720 – 0.963)	0.817 (0.699 – 0.935)
P10	0.878 (0.771 – 0.985)	0.834 (0.679 – 0.990)	0.839 (0.712 – 0.967)
P11	0.857 (0.758 – 0.956)	0.875 (0.762 – 0.988)	0.858 (0.755 – 0.960)
Mean	0.928 (0.865 – 0.991)	0.925 (0.864 – 0.986)	0.930 (0.874 – 0.985)
All	0.898 (0.786 – 0.991)	0.868 (0.737 – 0.991)	0.878 (0.749 – 0.990)

For all pathologists and all morphometric parameters significance ($p < 0.001$) was reached.

Mean, the mean of the measurements of the 9 pathologists per case was used to determine the AUC values.

All, combined data of all 9 pathologists (repeated measures) was used for analysis with bootstrapping.

SD, standard deviation

Supplemental Table S14. Area under the ROC curve (AUC) with 95% confidence intervals (95% CI) for the individual parameters of algorithmic nuclear morphometry regarding tumor-specific survival throughout the entire follow-up period. The 3-5 regions of interest (ROI) per outcome case were combined for this analysis. The mitotic count (benchmark) by the first pathologist archived an AUC of 0.885 (95%CI: 0.765 - 1.00, p < 0.001).

Morphometric feature	Morphometric parameter	AUC (95% CI)
Size (area in µm²)	Mean	0.921 (0.839 – 1.00) **
	Median	0.918 (0.832 – 1.00) **
	Standard deviation (SD)	0.943 (0.889 – 0.996) **
	90th percentile (90th P)	0.932 (0.862 – 1.00) **
	90th P/ median	0.846 (0.728 – 0.964) **
	Mean of the largest 10% of the nuclei	0.932 (0.864 – 1.00) **
	Percentage of large nuclei (>37.8 µm²)	0.925 (0.849 – 1.00) **
	Percentage of large nuclei (>50.3 µm²)	0.909 (0.815 – 1.00) **
	Skewness	0.665 (0.510 – 0.819) *
Shape	Mean eccentricity	0.471 (0.325 – 0.617)
	SD of eccentricity	0.825 (0.728 – 0.921) **
	Skewness eccentricity	0.615 (0.468 – 0.762)
	1 - Mean solidity	0.553 (0.390 – 0.706)
	SD of solidity	0.761 (0.641 – 0.881) **
	Percentage of nuclei with indentation (solidity <0.913)	0.757 (0.631 - 0.883) **
	Percentage of nuclei with indentation (solidity <0.936)	0.691 (0.555 - 0.828) *
	Percentage of nuclei with indentation (solidity < 0.943)	0.640 (0.497 - 0.784)

* p ≤ 0.05; ** p ≤ 0.001

Supplemental Table S15. Area under the ROC curve (AUC) with 95% confidence intervals (95% CI) for the individual parameters of algorithmic nuclear morphometry regarding tumor-specific survival at 12 months after surgery. Cases that had died within the first 12 months after surgery due to an ccMCT-unrelated cause (N = 6) were excluded for this analysis. The 3-5 regions of interest (ROI) per outcome case were combined for this analysis. The mitotic count (benchmark) by the first pathologist archived an AUC of 0.890 (95% CI: 0.797 – 0.982; p < 0.001).

**Morphometric feature**	**Morphometric parameter**	**AUC (95% CI)**
Size (area in µm²)	Mean	0.884 (0.737 – 0.992) **
	Median	0.862 (0.731 – 0.992) **
	Standard deviation (SD)	0.886 (0.780 – 0.992) **
	90th percentile (90th P)	0.881 (0.769 – 0.994) **
	90th P/ median	0.827 (0.690 – 0.965) **
	Mean of the largest 10% of the nuclei	0.873 (0.758 – 0.988) **
	Percentage of large nuclei (>37.8 µm²)	0.862 (0.729 – 0.995) **
	Percentage of large nuclei (>50.3 µm²)	0.808 (0.625 – 0.992) **
	Skewness	0.536 (0.354 – 0.718)
Shape	Mean eccentricity	0.495 (0.331 – 0.659)
	SD of eccentricity	0.750 (0.624 – 0.877) **
	Skewness eccentricity	0.589 (0.415 – 0.763)
	1 - Mean solidity	0.572 (0.400 – 0.743)
	SD of solidity	0.768 (0.661 – 0.874) **
	Percentage of nuclei with indentation (solidity <0.913)	0.741 (0.609 - 0.873) **

* p ≤ 0.05; ** p ≤ 0.001

Supplemental Table S16. Area under the ROC curve (AUC) with 95% confidence intervals (95% CI) for the individual parameters of algorithmic nuclear morphometry regarding overall mortality at 12 months after surgery (cases with death within the first 12 months after surgery regardless of cause compared to cases that survived for at least 12 months). The 3-5 regions of interest (ROI) per outcome case were combined for this analysis. The mitotic count (benchmark) by the first pathologist archived an AUC of 0.744 (95% CI: 0.596 – 0.891, p = 0.001).

Morphometric feature	Morphometric parameter	AUC (95% CI)
Size (area in µm²)	Mean	0.730 (0.568 – 0.893) *
	Median	0.723 (0.558 – 0.888) *
	Standard deviation (SD)	0.766 (0.618 – 0.914) **
	90th percentile (90th P)	0.749 (0.593 – 0.906) *
	90th P / median	0.696 (0.524 – 0.869) *
	Mean of the largest 10% of the nuclei	0.744 (0.590 – 0.897) *
	Percentage of large nuclei (>37.8 µm²)	0.755 (0.602 – 0.907) **
	Percentage of large nuclei (>50.3 µm²)	0.738 (0.590 – 0.887) *
	Skewness	0.524 (0.379 – 0.669)
Shape	Mean eccentricity	0.441 (0.272 – 0.610)
	SD of eccentricity	0.606 (0.452 – 0.760)
	Skewness eccentricity	0.630 (0.459 – 0.800)
	1 - Mean solidity	0.458 (0.401 – 0.719)
	SD of solidity	0.691 (0.558 – 0.825) *
	Percentage of nuclei with indentation (solidity <0.913)	0.700 (0.560 - 0.840) *

* p ≤ 0.05; ** p ≤ 0.001

Scatter plots

Gold Standard Manual Nuclear Morphometry (≥100 nuclei)

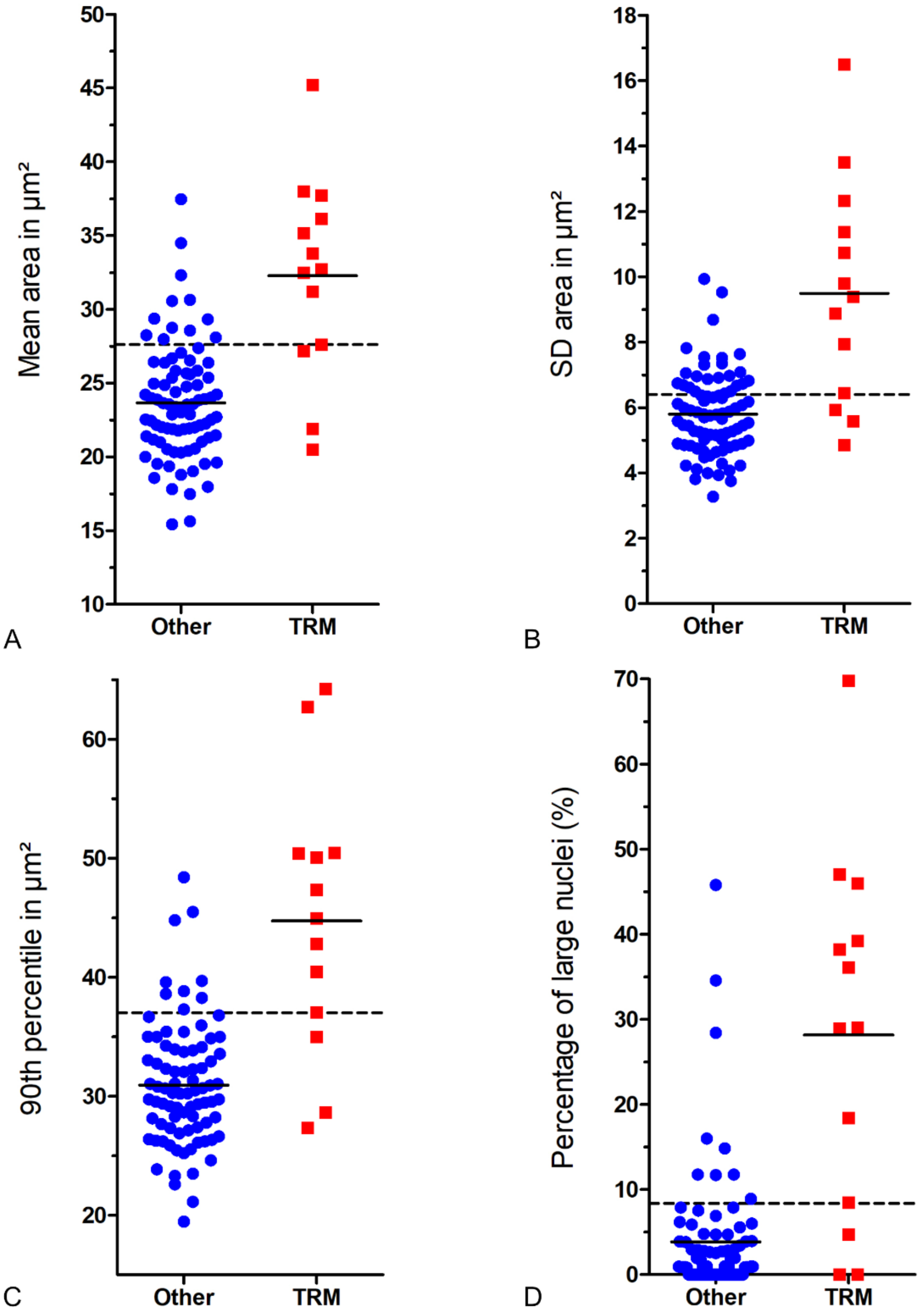

Supplemental Figure S14. Scatterplots for area parameters of gold standard manual nuclear morphometry (≥100 nuclei) comparing cases with tumor-related mortality (TRM) with others (survived observational period or died due to tumor-unrelated cause). The broken line represents threshold 1. The short solid lines represent the means of the measurement of the respective outcome group. **A)** Mean nuclear area in µm², **B)** standard deviation (SD) of nuclear area in µm², **C)** 90th percentile of nuclear area in µm², and **D)** percentage (%) of large nuclei with an area >37.8 µm².

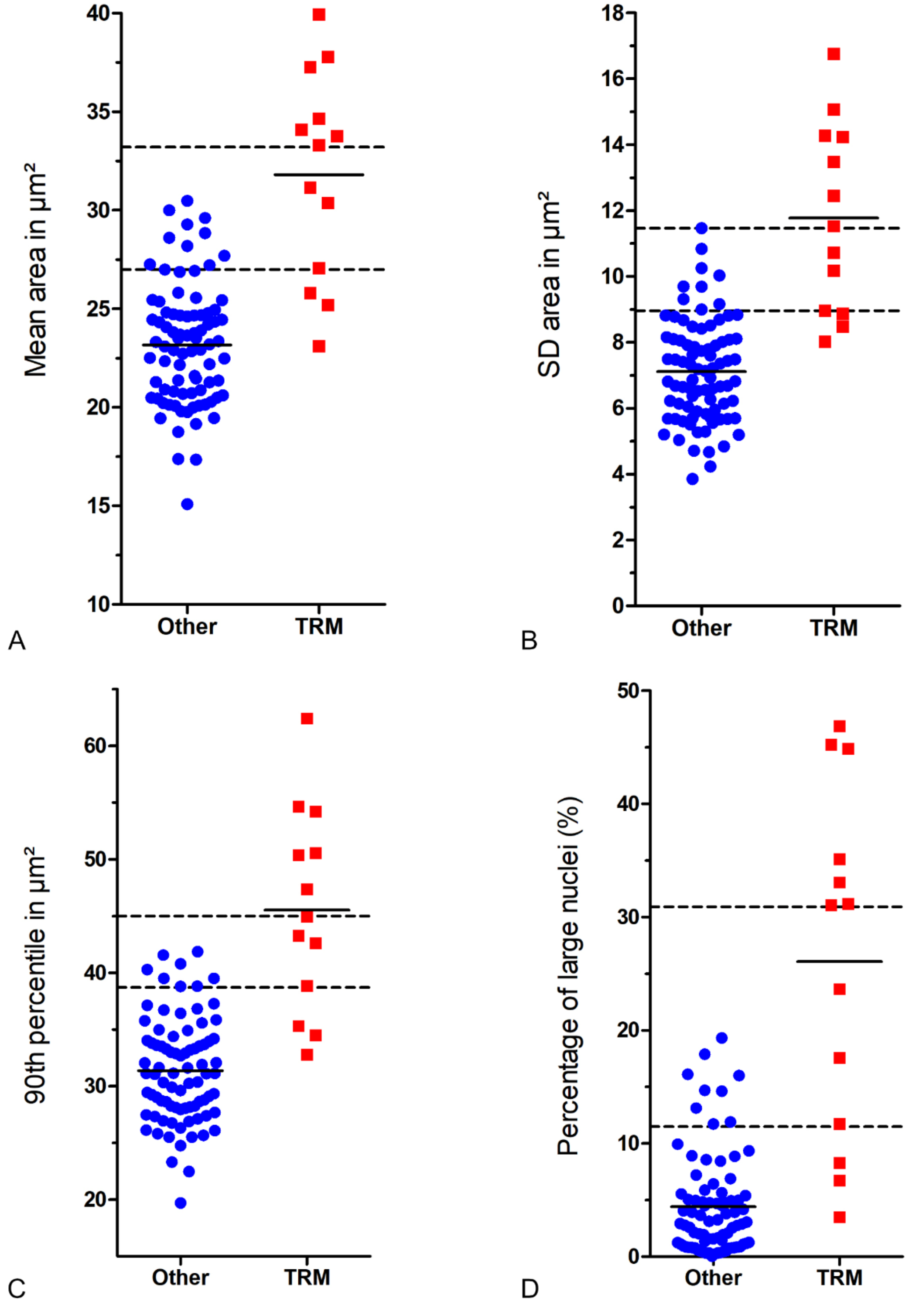

Supplemental Figure S15. Scatterplots for area parameters of algorithmic nuclear morphometry comparing cases with tumor-related mortality (TRM) with others (survived observational period or died due to tumor-unrelated cause). The lower broken line represents threshold 1 and the upper broken line represents threshold 2. The short solid lines represent the means of the measurement of the respective outcome group. **A)** Mean nuclear area in µm², **B)** standard deviation (SD) of nuclear area in µm², **C)** 90th percentile of nuclear area in µm², and **D)** percentage (%) of large nuclei with an area >37.8 µm².

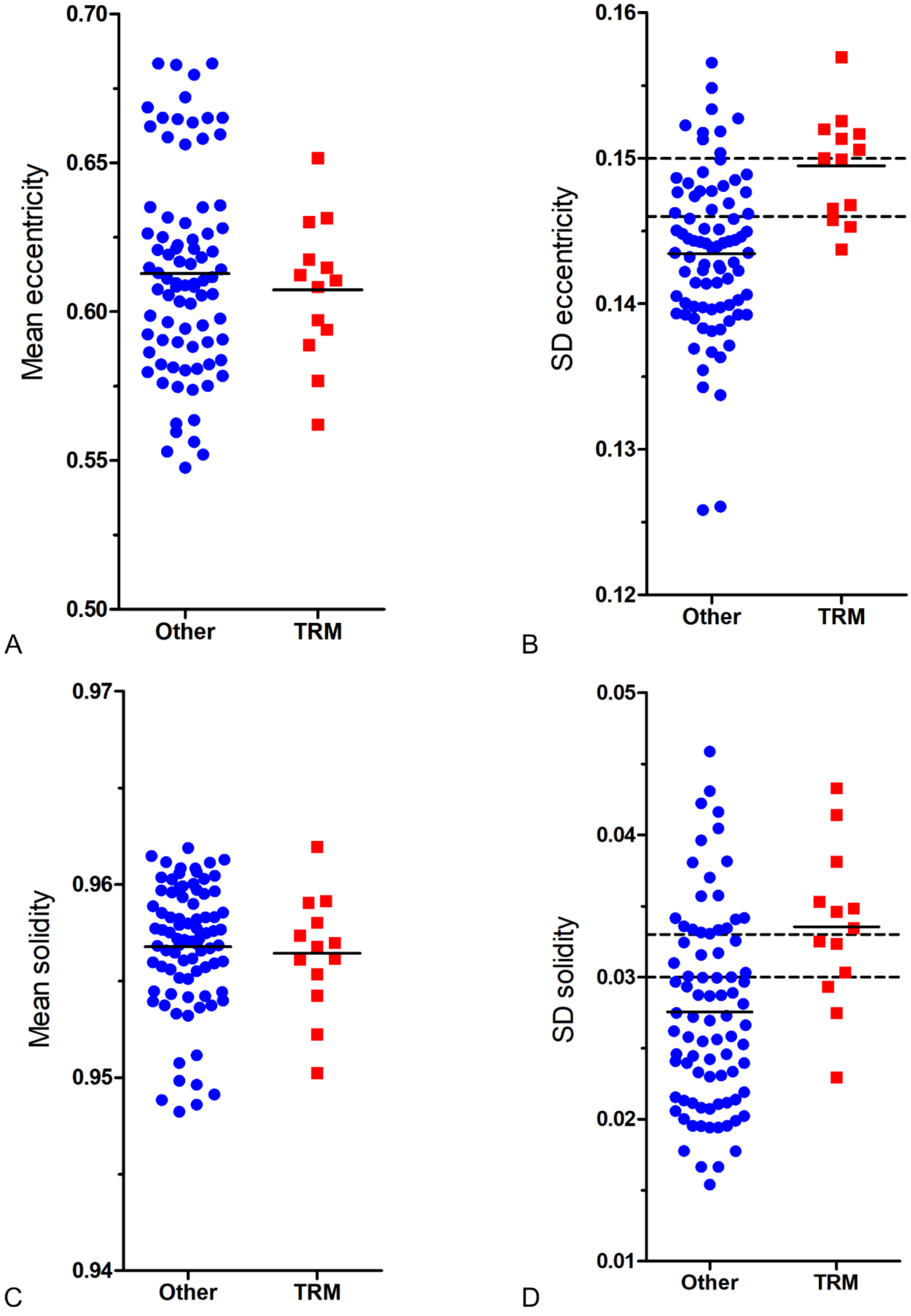

Supplemental Figure S16. Scatterplots for shape parameters of <u>algorithmic nuclear morphometry</u> comparing cases with tumor-related mortality with others (survived observational period or died due to tumor-unrelated cause). The lower broken line represents threshold 1 and the upper broken line represents threshold 2. The short solid lines represent the means of the measurement of the respective outcome group. **A)** Mean eccentricity **B)** SD of eccentricity, **C)** mean solidity, and **D)** SD of eccentricity.

Sensitivity and Specificity

Pathologists' Estimates

Supplemental Table S17. Sensitivity (Sen), specificity (Sp), and precision (Pre, also known as positive predictive value) regarding tumor-specific mortality for three-tier anisokaryosis estimates combining two of the three categories (1 = no to mild, 2 = moderate, 3 = severe).

Path-ologist	Time point	Anisokaryosis 1 vs. 2+3			Anisokaryosis 1+2 vs. 3		
		Sen	Sp	Pre	Sen	Sp	Pre
P1	1	76.9%	85.5%	45.5%	0%	100%	NA
	2	61.5%	90.4%	50.0%	0%	100%	NA
P2	1	76.9%	48.2%	18.9%	38.5%	96.4%	62.5%
	2	69.2%	71.1%	27.3%	23.1%	95.2%	42.9%
P3	1	92.3%	49.4%	22.2%	46.2%	88.0%	37.5%
	2	92.3%	32.5%	17.6%	76.9%	84.3%	43.5%
P4	1	92.3%	51.8%	23.1%	61.5%	96.4%	72.7%
P5	1	84.6%	38.6%	17.7%	38.5%	86.7%	31.3%
	2	92.3%	19.3%	15.2%	84.6%	69.9%	30.6%
P6	1	69.2%	54.2%	19.1%	46.2%	95.2%	60.0%
	2	76.9%	60.2%	23.3%	30.8%	98.8%	80.0%
P7	1	46.2%	98.8%	85.7%	0%	100%	NA
	2	61.5%	89.2%	47.1%	0%	100%	NA
P8	1	100%	13.3%	15.3%	69.2%	90.4%	52.9%
	2	100%	4.8%	14.1%	76.9%	77.1%	34.5%
P9	1	46.2%	97.6%	75.0%	7.7%	100%	100%
P10	1	76.9%	66.3%	26.3%	53.8%	92.8%	53.8%
	2	53.8%	89.2%	43.8%	23.1%	98.8%	75.0%
P11	1	76.9%	78.3%	35.7%	30.8%	98.8%	80.0%
	2	61.5%	81.9%	34.8%	30.8%	100%	100%

NA, not available due to division by 0.

Supplemental Table S18. Sensitivity, specificity, and precision (also known as positive predictive value) regarding tumor-specific mortality for karyomegaly estimates.

Pathologist	Time point	Karyomegaly (presence vs. absence)		
		Sensitivity	Specificity	Precision
P1	1	38.5%	95.2%	55.6%
	2	15.4%	98.8%	66.7%
P2	1	61.5%	71.1%	25.0%
	2	46.2%	92.8%	50.0%
P3	1	30.8%	100%	100%
	2	38.5%	95.2%	55.6%
P4	1	46.2%	97.6%	75.0%
P5	1	0%	98.8%	0%
	2	30.8%	94.0%	44.4%
P6	1	69.2%	66.3%	24.3%
	2	69.2%	75.9%	31.0%
P7	1	23.1%	98.8%	75.0%
	2	23.1%	100%	100%
P8	1	69.2%	97.6%	81.8%
	2	69.2%	95.2%	69.2%
P9	1	76.9%	83.1%	41.7%
P10	1	76.9%	65.1%	25.6%
	2	53.8%	89.2%	43.8%
P11	1	92.3%	32.5%	17.6%
	2	92.3%	31.3%	17.4%
Majority vote	1	61.5%	97.6%	80.0%
	2	38.5%	98.8%	83.3%

Majority vote, the karyomegaly category that was given by at least 6/11 (time point 1) pathologists or by at least 5/9 (time point 2) pathologists.

Supplemental Table S19. Sensitivity, specificity, and precision (also known as positive predictive value) regarding tumor-related mortality for the different morphometric parameters of gold standard manual nuclear morphometry (≥100 nuclei) using threshold 1. For standard deviation (SD) of area two further threshold are applied (9.3 µm² represents threshold 2).

**Morphometric parameter**	**Threshold value**	**Sensitivity**	**Specificity**	**Precision**
Mean area	27.6 µm²	76.9%	85.5%	45.5%
Median area	26.8 µm²	76.9%	83.1%	41.7%
SD of area	6.4 µm²	76.9%	71.3%	29.4%
	7.9 µm²	69.2%	96.4%	75.0%
	9.3 µm²	53.8%	97.6%	77.8%
90th percentile (90th P)	37.0 µm²	76.9%	89.2%	52.6%
Mean of the largest 10% of the nuclei	40.3 µm²	76.9%	85.5%	45.5%
Percentage of large nuclei (>37.8 µm²)	8.4%	76.9%	85.5%	45.5%
Percentage of large nuclei (>50.3 µm²)	0.97%	76.9%	89.2%	52.6%
SD of solidity	0.013	76.9%	73.5%	31.3%

Supplemental Table S20. Sensitivity (Sen) and specificity (Sp) regarding tumor-specific mortality for the different morphometric parameters of practicable manual nuclear morphometry (12 nuclei) using threshold 1. The same threshold (thres.) per parameter was applied to all pathologists and was calculated from the mean pathologists' measurements of the 96 outcome cases.

**Path-ologist**	**Mean area (thres.: 33.4 µm²)**		**SD of area (thres.: 10.9 µm²)**		**Maximum area (thres.: 54.9 µm²)**	
	**Sen**	**Sp**	**Sen**	**Sp**	**Sen**	**Sp**
P1	84.6%	91.6%	61.5%	91.6%	69.2%	90.4%
P2	84.6%	79.5%	53.8%	84.3%	84.6%	88.0%
P3	38.5%	98.8%	76.9%	88.0%	53.8%	95.2%
P5	53.8%	92.8%	76.9%	72.3%	76.9%	85.5%
P6	53.8%	97.6%	46.2%	80.7%	46.2%	89.2%
P7	84.6%	71.1%	76.9%	92.8%	92.3%	88.0%
P8	69.2%	84.3%	69.2%	85.5%	61.5%	89.2%
P10	76.9%	79.5%	84.6%	79.5%	84.6%	83.1%
P11	100%	50.6%	84.6%	78.3%	76.9%	71.1%
Mean	76.9%	88.0%	76.9%	85.5%	76.9%	89.2%

Mean, the mean of the measurement of the 9 pathologists per case was used to determine the sensitivity and specificity values.

SD, standard deviation

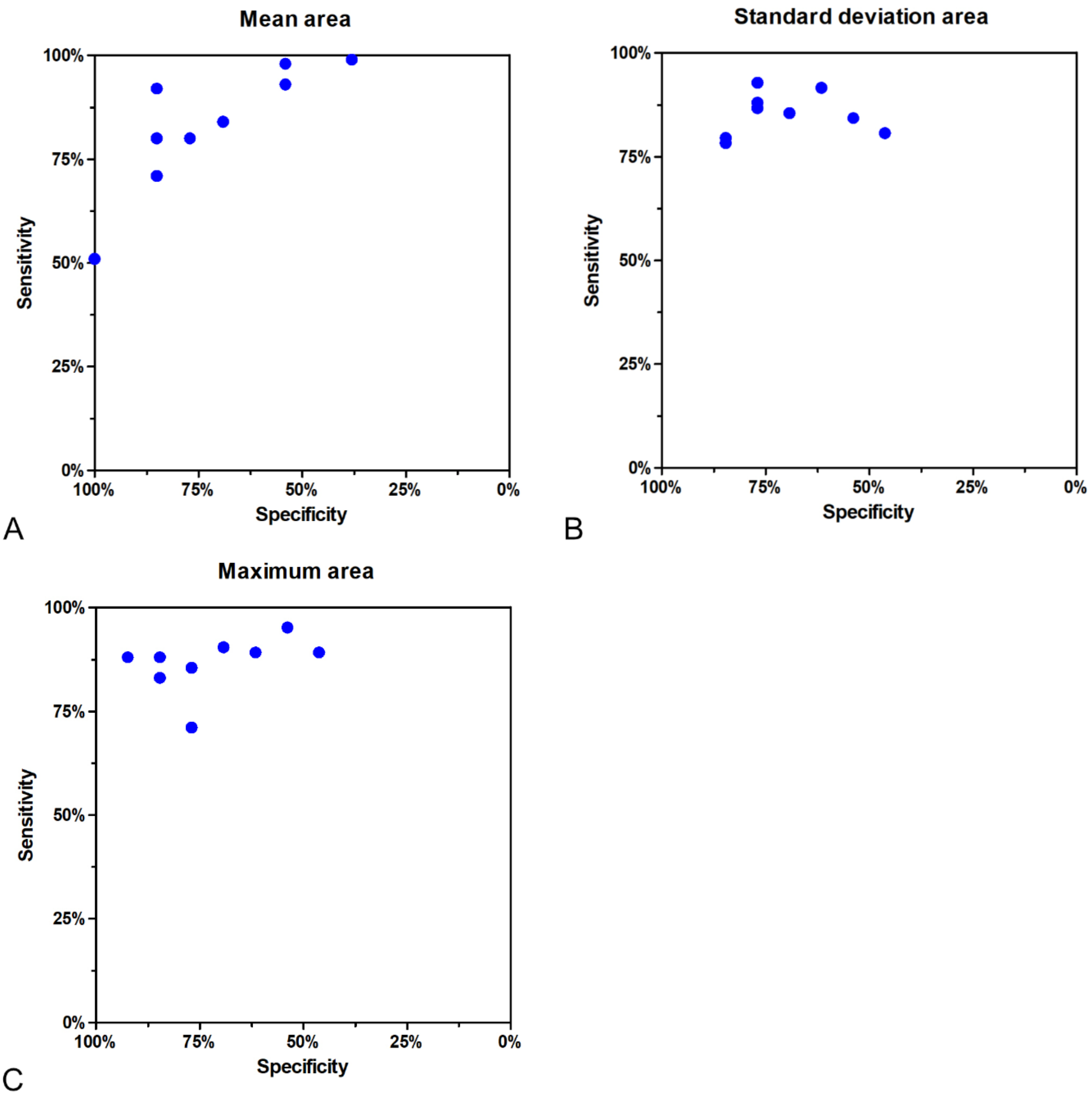

Supplemental Figure S17. Graphical comparison of the sensitivity and specificity values regarding tumor-related mortality for the different size parameters of practicable manual nuclear morphometry (12 nuclei). Each datapoint (blue dots) represents an individual pathologist. For case classification threshold 1 (based on the mean measurement of the 9 pathologists) was used. **A)** Mean area (threshold: 33.4 µm²), **B)** standard deviation of nuclear area (threshold: 10.9 µm²), and **C)** maximum area (threshold: 54.9 µm²).

Supplemental Table S21. Sensitivity, specificity, and precision (also known as positive predictive value) regarding tumor-related mortality for the different morphometric parameters of algorithmic nuclear morphometry using threshold 1.

Morphometric parameter	Threshold value	Sensitivity	Specificity	Precision
Mean area	27.0 µm²	76.9%	86.7%	47.6%
Median area	27.5 µm²	76.9%	91.6%	58.8%
SD of area	9.0 µm²	76.9%	89.2%	52.6%
90th percentile (90th P)	38.7 µm²	76.9%	90.4%	55.6%
90th P / median	1.40	76.9%	79.5%	37.0%
Mean of the largest 10% of the nuclei	44.1 µm²	76.9%	85.5%	45.5%
Percentage of large nuclei (>37.8 µm²)	11.5 %	76.9%	89.2%	52.6%
Percentage of large nuclei (>50.3 µm²)	2.4 %	76.9%	90.4%	55.6%
SD of eccentricity	0.146	76.9%	69.9%	28.6%
SD of solidity	0.030	76.9%	68.7%	27.8%

SD, standard deviation

Supplemental Table S22. Sensitivity, specificity, and precision (also known as positive predictive value) regarding tumor-related mortality for the different morphometric parameters of algorithmic nuclear morphometry using threshold 2.

Morphometric parameter	Threshold value	Sensitivity	Specificity	Precision
Mean area	33.2 µm²	53.8%	100%	100%
Median area	31.7 µm²	53.8%	100%	100%
SD of area	11.5 µm²	53.8%	100%	100%
90th percentile (90th P)	45.0 µm²	53.8%	100%	100%
90th P / median	1.44	53.8%	90.4%	46.7%
Mean of the largest 10% of the nuclei	53.9 µm²	53.8%	100%	100%
Percentage of large nuclei (>37.8 µm²)	31.0 %	53.8%	100%	100%
Percentage of large nuclei (>50.3 µm²)	4.9 %	53.8%	100%	100%
SD of eccentricity	0.150	53.8%	89.2%	43.8%
SD of solidity	0.033	53.8%	78.3%	28.0%

SD, standard deviation

Kaplan-Meier Curves

Pathologists' Estimates

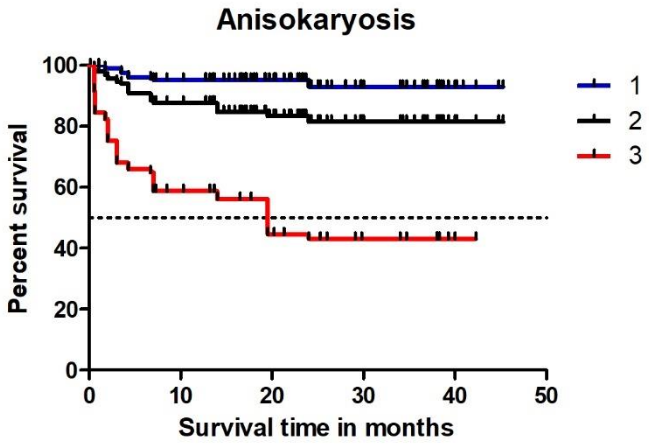

Supplemental Figure S18. Kaplan-Meier curves regarding tumor-specific survival time for three-tier anisokaryosis estimates (combined data from time point 1 of all 11 pathologists). Compared to category 1 (none to mild) as a reference, the hazard ratio of category 2 (moderate) is 5.0 (95% CI: 3.5 – 7.0, $p < 0.001$) and for category 3 (severe) is 23.6 (95% CI: 16.1 – 34.2, $p < 0.001$).

Gold Standard Manual Nuclear Morphometry (≥100 nuclei)

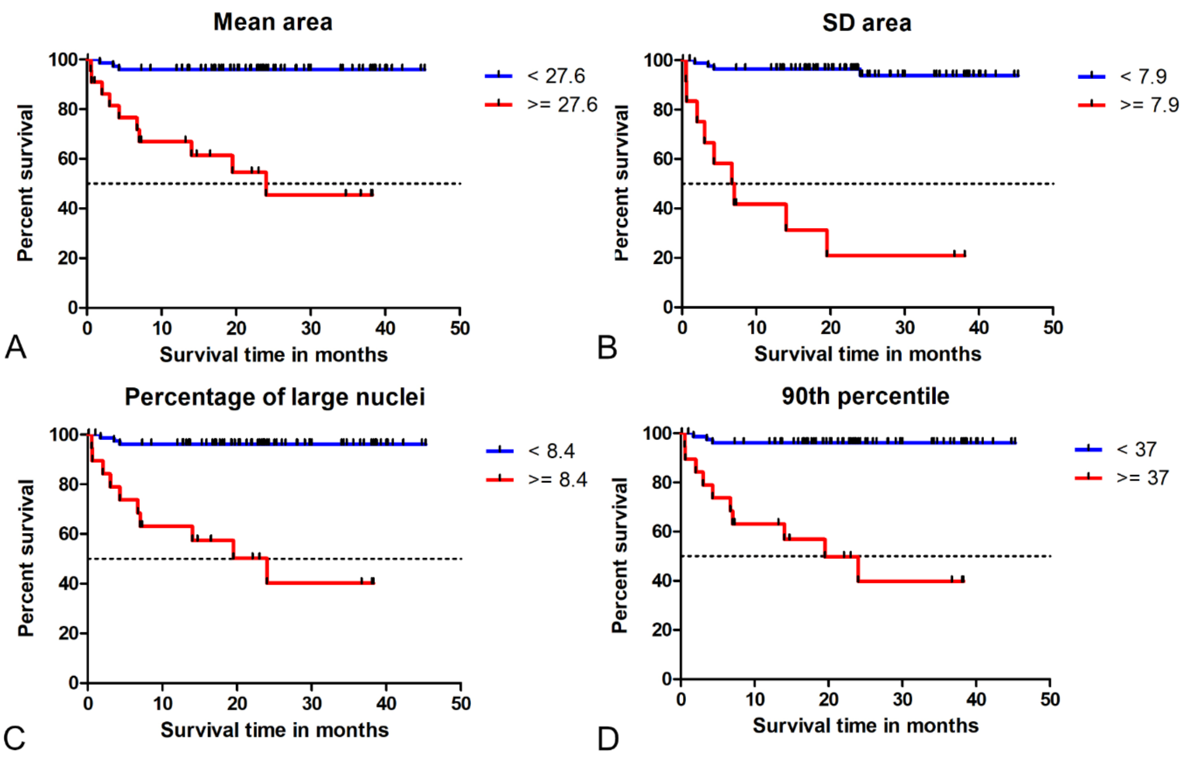

Supplemental Figure S19. Kaplan-Meier curves and log rank tests (p values below) regarding tumor-specific survival time for different size parameters of gold standard manual nuclear morphometry (≥100 nuclei). Cases are classified by threshold 1. **A)** Mean nuclear area ($p < 0.001$), **B)** standard deviation (SD) of nuclear area ($p < 0.001$), **C)** 90th percentile of nuclear area ($p < 0.001$), and **D)** percentage of large nuclei with an area >37.8 µm² ($p < 0.001$).

Practicable Manual Nuclear Morphometry (12 nuclei)

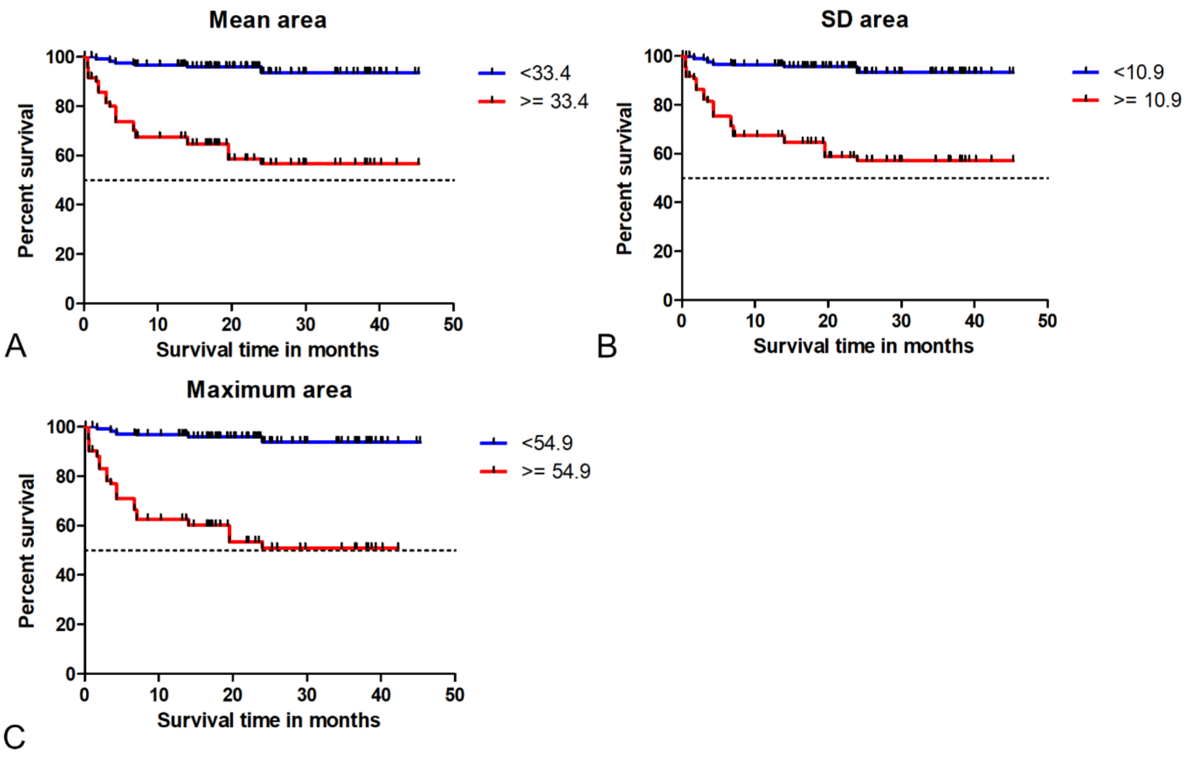

Supplemental Figure S20. Kaplan-Meier curves regarding tumor-specific survival time for different size parameters of practicable manual nuclear morphometry (12 nuclei). The combined data of all 9 pathologists were used and the log rank test is not provided due to repeated measures data; for results on statistical significance see hazard ratios below. Cases are classified by threshold 1 based on the mean measurements of the pathologists. **A)** Mean area, **B)** standard deviation (SD) of area, and **C)** maximum area.

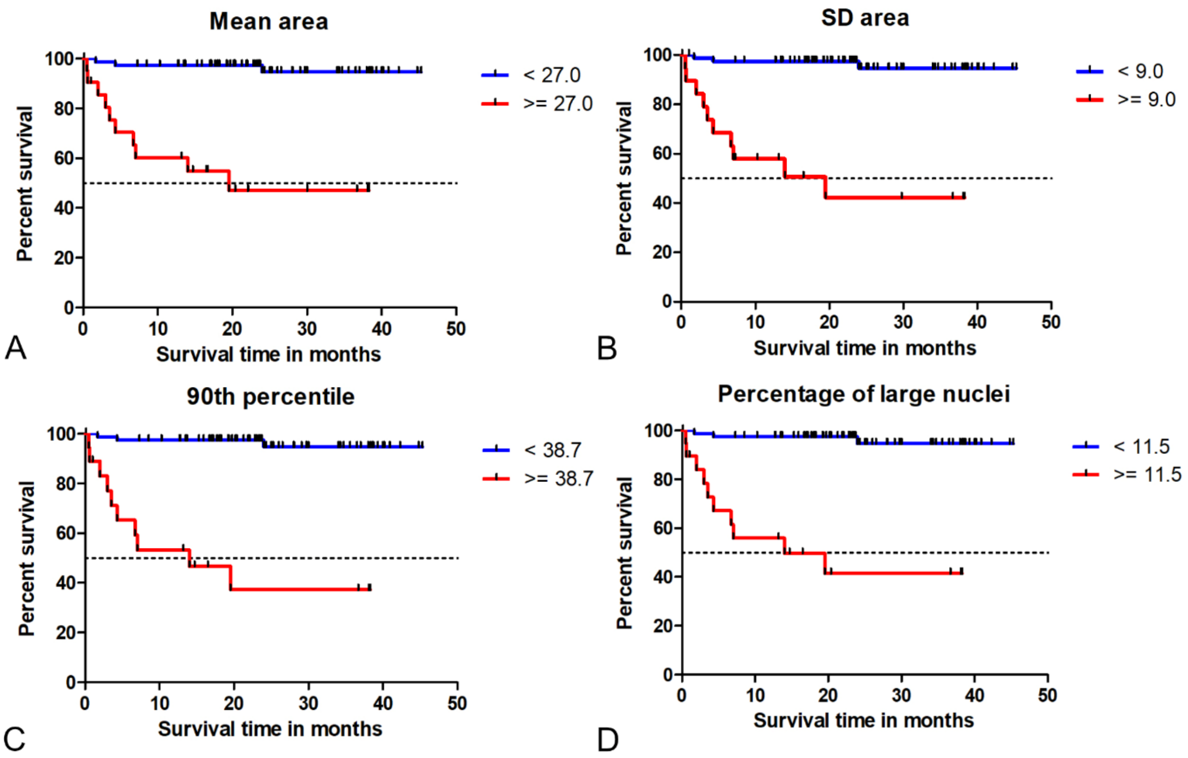

Supplemental Figure S21. Kaplan-Meier curves and log rank tests (p values below) regarding tumor-specific survival time for different size parameters of algorithmic nuclear morphometry. Cases are classified by threshold 1. **A)** Mean nuclear area ($p < 0.001$), **B)** standard deviation (SD) of nuclear area ($p < 0.001$), **C)** 90th percentile of nuclear area ($p < 0.001$), and **D)** percentage of large nuclei with an area >37.8 µm² ($p < 0.001$).

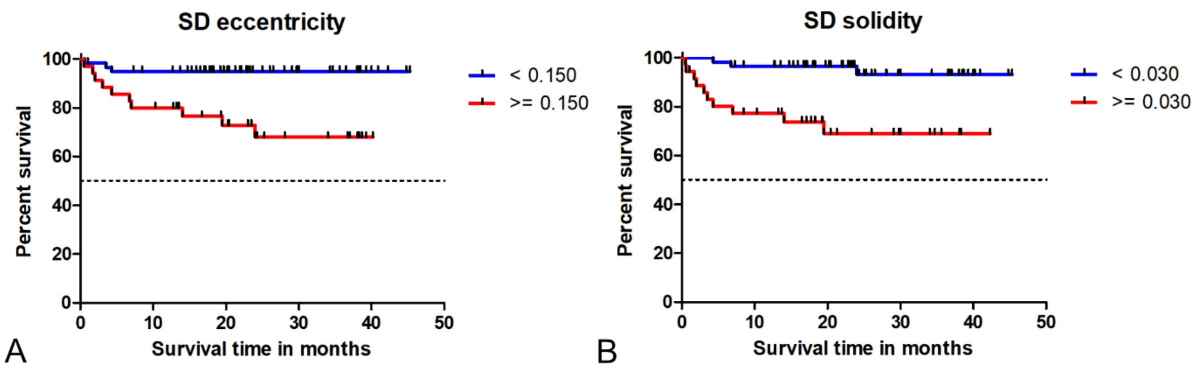

Supplemental Figure S22. Kaplan-Meier curves and log rank tests (p values below) regarding tumor-specific survival time for different shape parameters of algorithmic nuclear morphometry. Cases are classified by threshold 1. **A)** Standard deviation (SD) of eccentricity ($p = 0.0018$), **B)** SD of solidity ($p < 0.001$).

Mitotic Count

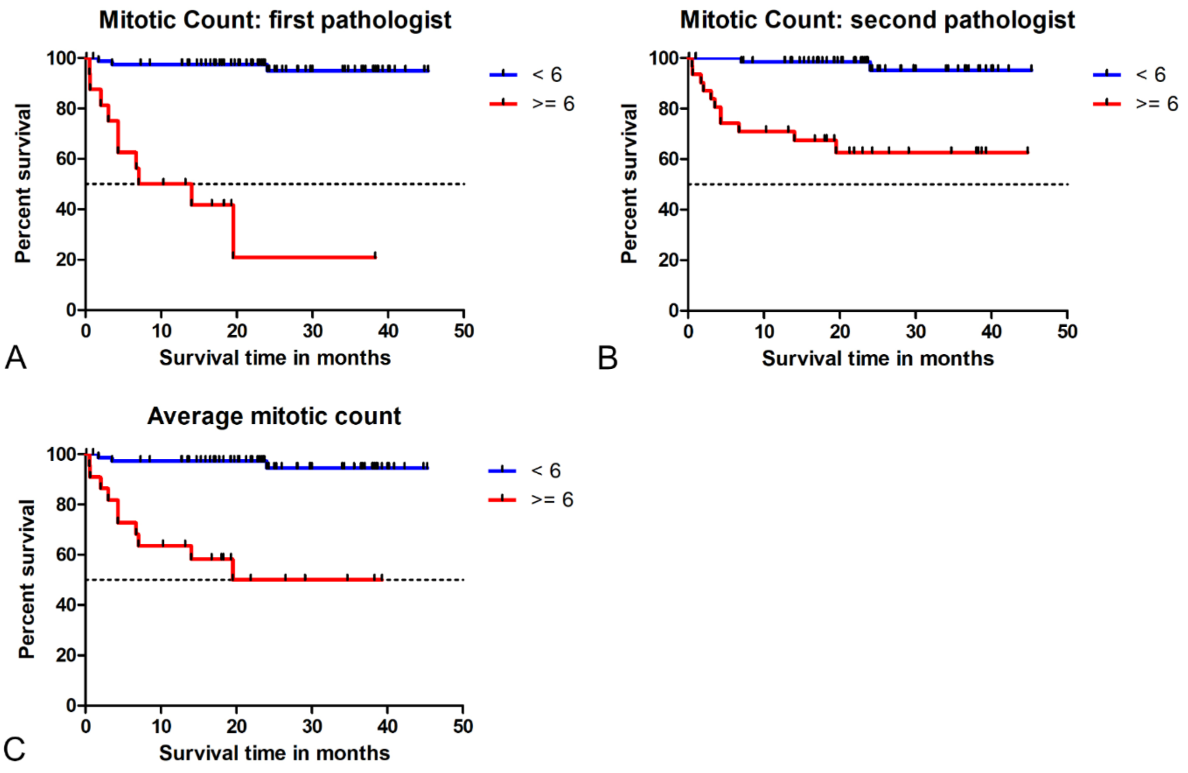

Supplemental Figure S23. Kaplan-Meier curve regarding tumor-specific survival for the mitotic count (MC). Cases are classified by the threshold proposed by Romansik et al. (Vet Pathol, 2007; DOI: 10.1354/vp.44-3-335). A) MC by the first pathologist, log rank test: $p < 0.001$. B) MC by the second pathologist, log rank test: $p < 0.001$. C) Average MC of both pathologist, log rank test: $p < 0.001$.

Hazard Ratios (Univariate Cox Regression with Categorical Data)

Pathologists' Estimates

Supplemental Table S23. Hazard ratios with 95% confidence intervals determined from univariate cox regression regarding tumor-specific survival time for the three-tier anisokaryosis (estimates combined to two categories; 1 = no to mild, 2 = moderate, 3 = severe) and karyomegaly (presence vs. absence) estimates from time point 1.

**Path-ologist**	**Three-tier anisokaryosis**		**Karyomegaly**
	**1 vs. 2+3**	**1+2 vs. 3**	
P1	15.4 (4.2 – 56.4) **	NA	8.3 (2.7 – 25.8) **
P2	3.0 (0.82 – 10.9)	11.4 (3.7 – 35.4) **	3.6 (1.16 – 10.9) *
P3	10.1 (1.31 – 77.9) *	5.2 (1.74 – 15.5) *	19.5 (5.8 – 65.5) **
P4	11.9 (1.54 – 91.4) *	21.5 (6.9 – 66.6) **	15.7 (5.1 – 47.5) **
P5	3.1 (0.69 – 14.0)	3.3 (1.07 – 10.1) *	0.49 (0 – ND)
P6	2.4 (0.74 – 7.8)	11.1 (3.6 – 33.2) **	3.7 (1.15 – 12.1) *
P7	24.2 (7.6 – 76.8) **	NA	12.9 (3.4 – 48.3) **
P8	25.6 (0.26 – ND)	13.5 (4.1 – 44.2) **	27.5 (8.3 – 90.3) **
P9	16.3 (5.4 – 49.3) **	7.6 (0.98 – 58.8)	12.7 (3.4 – 46.4) **
P10	5.9 (1.61 – 21.4) *	11.2 (3.7 – 33.9) **	5.6 (1.5 – 20.2) *
P11	10.3 (2.8 – 37.5) **	11.6 (3.5 – 37.8) **	5.5 (0.71 – 42.6)
Majority vote	Not evaluated	Not evaluated	23.8 (7.6 – 74.7) **

* $p \leq 0.05$; ** $p \leq 0.001$

ND, not determined as statistical model did not converge

NA, not available as no category 3 anisokaryosis was determined by this pathologist.

Majority vote, the karyomegaly category that was given by at least 6/11 pathologists

Supplemental Table S24. Hazard ratios (HR) with 95% confidence intervals (95% CI) determined from univariate cox regression regarding tumor-specific survival time for the different morphometric parameters of gold standard manual nuclear morphometry (≥100 nuclei). Cases are dichotomized based on threshold 1.

**Morphometric category**	**Morphometric parameter**	**Threshold 1**	**HR (95% CI)**
Size (area in µm²)	Mean	27.6 µm²	14.4 (3.9 – 52.3) **
	Median	26.8 µm²	12.8 (3.5 – 46.6) **
	Standard deviation (SD)	6.4 µm²	6.2 (1.7 – 22.5) *
		7.9 µm²	24.8 (7.5 – 81.2) **
	90th percentile (90th P)	37.0 µm²	16.9 (4.6 – 61.7) **
	Mean of the largest 10% of the nuclei	40.3 µm²	13.2 (3.6 – 47.9) **
	Percentage of large nuclei (>37.8 µm²)	8.4%	16.8 (4.6 – 61.2) **
	Percentage of large nuclei (>50.3 µm²)	0.97%	9.4 (2.8 – 30.7) **
Shape	SD of solidity	0.013	7.3 (2.0 – 26.8) *

* p ≤ 0.05; ** p ≤ 0.001

Supplemental Table S25. Hazard ratios (HR) with 95% confidence intervals determined from univariate Cox regression regarding tumor-specific survival time for the different morphometric area parameters of manual nuclear morphometry (12 nuclei). Cases are dichotomized by threshold 1 (thes., based on the mean measurements of the pathologists).

Path-ologist	Mean area (thres.: 33.4 µm²)	SD of area (thres.: 10.9 µm²)	Maximum area (thres.: 54.9 µm²)
P1	35.4 (7.7 – 160.9) **	12.5 (4.0 – 38.8) **	15.8 (4.7 – 51.9) **
P2	15.7 (3.4 – 71.0) **	4.8 (1.6 – 14.2) *	24.1 (5.3 – 108.9) **
P3	18.8 (6.0 – 58.6) **	15.0 (4.1 – 54.7) **	13.6 (4.5 – 40.7) **
P5	10.5 (3.5 – 31.5) **	6.9 (1.8 – 25.0) *	13.0 (3.5 – 47.2) **
P6	19.9 (6.5 – 60.0) **	3.3 (1.1 – 9.8) *	5.5 (1.86 – 16.6) *
P7	11.5 (2.5 – 51.8) *	27.8 (7.5 – 102.7) **	63.1 (8.1 – 488.7) **
P8	9.4 (2.8 – 30.5) **	9.2 (2.8 – 29.7) **	8.8 (2.8 – 27.0) **
P10	10.6 (2.8 – 38.6) **	15.0 (3.3 – 67.6) **	18.7 (4.1 – 84.5) **
P11	56.6 (0.64 – ND)	15.0 (3.3 – 67.6) **	6.9 (1.90 – 25.3) *
Mean	17.1 (4.7 – 62.6) **	13.1 (3.6 – 47.6) **	16.9 (4.6 – 61.5) **
All	13.7 (8.9 – 21.2) **	9.0 (6.0 – 13.4) **	12.0 (8.0 – 18.0) **

* $p \leq 0.05$; ** $p \leq 0.001$

SD, standard deviation

ND, not determined as statistical model did not converge

Mean, the mean of the measurements of the 9 pathologists per case was used to determine the HR values.

All, combined data of all 9 pathologists (repeated measures) was used for analysis with a mixed model.

Supplemental Table S26. Hazard ratios (HR) with 95% confidence intervals (95% CI) determined from univariate cox regression regarding tumor-specific survival time for the different morphometric parameters of algorithmic nuclear morphometry. Cases are dichotomized based on threshold 1.

**Morphometric category**	**Morphometric parameter**	**Threshold 1**	**HR (95% CI)**
Size (area in µm²)	Mean	27.0 µm²	16.8 (4.5 – 61.4) **
	Median	27.5 µm²	23.2 (6.3 – 85.0) **
	Standard deviation (SD)	9.0 µm²	18.3 (5.0 – 67.1) **
	90th percentile (90th P)	38.7 µm²	21.4 (5.8 – 78.5) **
	90th P/ median	1.40	10.5 (2.8 – 38.4) **
	Mean of the largest 10% of the nuclei	44.1 µm²	14.8 (4.0 – 54.1) **
	Percentage of large nuclei (>37.8 µm²)	11.5 %	19.5 (5.3 – 71.4) **
	Percentage of large nuclei (>50.3 µm²)	2.4 %	19.6 (5.3 – 71.9) **
Shape	SD of eccentricity	0.146	6.1 (1.6 – 22.1) *
	SD of solidity	0.030	6.7 (1.8 – 24.5) *

* p ≤ 0.05; ** p ≤ 0.001

Heterogeneity between tumor regions

Supplemental Table S27. Summary of the coefficient of variation (standard deviation / mean) for the measurements in the 3-5 regions of interest (ROIs) comparing the different parameters of algorithmic morphometry.

Category	Morphometric parameter	Coefficient of variation	
		**Mean of all cases**	**Range**
Size (area in µm²)	Mean	8.3%	1.4 – 17.5%
	Standard deviation (SD)	13.3%	2.2 – 30.5%
	90th percentile	8.9%	1.2 – 21.1%
	Mean of the largest 10% of the nuclei	10.4%	1.6 – 23.2%
	Percentage of large nuclei (>37.8 µm²)	65.2% *	6.9% - 164.5% *
Shape	SD of eccentricity	4.2%	0.6 – 11.0%
	SD of solidity	17.2%	3.0 – 17.2%

* The high coefficient of variation is mostly caused by absolute differences of a few percentages between the ROIs in cases with low values (<5%) on average, resulting in high relative difference for the coefficient of variation.

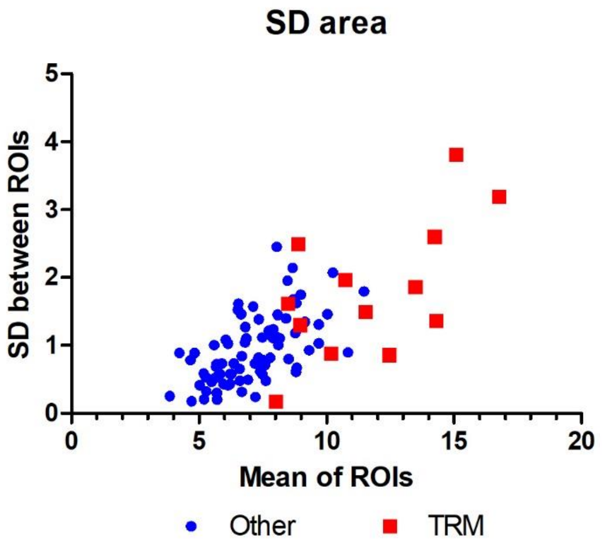

Supplemental Figure S24. Scatterplot for algorithmic morphometry of the standard deviation of the nuclear area comparing the mean values of all regions of interest (ROIs) per case and the standard deviation (SD) between the 5 tumor ROIs. The red squares represent cases with tumor-related mortality (TRM) throughout the entire follow-up period and the blue dots represent cases without TRM. This graph shows that the variability between the tumor regions (ROIs) of a case increases with its overall (mean) higher SD of nuclear area.

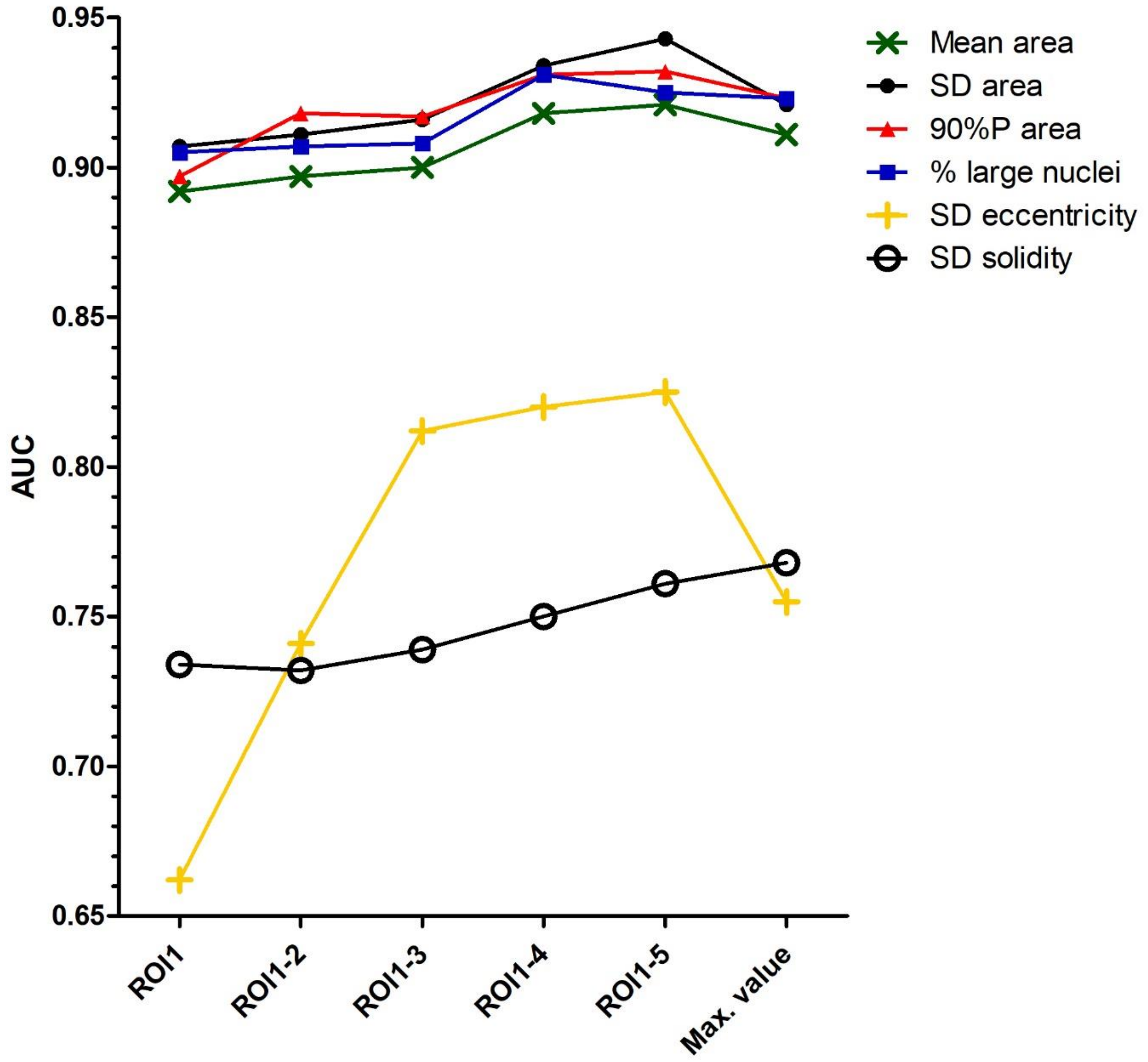

Supplemental Figure S25. Line diagram of the area under the curve (AUC) values regarding tumor-related mortality (throughout the entire follow-up period) for different parameters of algorithmic morphometry comparing the number of tumor regions of interest (ROI) used for statistical analysis. An increasing number of ROIs (1-5 according to their order of selection) was included. The maximum (max.) value is the measurement of the one ROI with the highest measurement of the 5 ROIs per case.

Supplemental Table S28. Area under the ROC curve values for tumor heterogeneity between the 3-5 ROIs per case as a prognostic test for tumor-specific survival using different parameters of algorithmic morphometry.

**Morphometric parameter**	**Area under the curve (95% confidence interval)**	
	**SD between ROIs**	**Proportion of hotspots**
Mean area	0.606 (0.415 – 0.798)	0.890 (0.780 – 0.999) **
SD of area	0.790 (0.632 – 0.947) **	0.890 (0.780 – 1.00) **
90th percentile	0.696 (0.521 – 0.871) *	0.918 (0.817 – 1.00) **
Mean of the largest 10% of the nuclei	0.759 (0.602 – 0.916) **	0.899 (0.789 – 1.00) **
Percentage of large nuclei (>37.8 µm²)	0.867 (0.760 – 0.975) **	0.911 (0.809 – 1.00) **
SD of eccentricity	0.554 (0.390 – 0.718)	0.814 (0.722 – 0.905) **
SD of solidity	0.683 (0.545 – 0.821) *	0.739 (0.623 – 0.855) **

ROIs, regions of interest locations in the tumor; hotspot, ROI with a morphometric measurement above threshold 1; SD, standard deviation; *, p ≤ 0.05; **, p ≤ 0.001
